# Longitudinal copy number, whole exome and targeted deep sequencing of 'good risk' IGHV-mutated CLL patients with progressive disease

**DOI:** 10.1038/leu.2016.10

**Published:** 2016-02-26

**Authors:** M J J Rose-Zerilli, J Gibson, J Wang, W Tapper, Z Davis, H Parker, M Larrayoz, H McCarthy, R Walewska, J Forster, A Gardiner, A J Steele, C Chelala, S Ennis, A Collins, C C Oakes, D G Oscier, J C Strefford

**Affiliations:** 1 Academic Unit of Cancer Sciences, Faculty of Medicine, University of Southampton, Southampton, UK; 2 Centre for Biological Sciences, Faculty of Natural and Environmental Studies, University of Southampton, Southampton, UK; 3 Bioinformatics Unit, Barts Cancer Institute, Barts and the London School of Medicine and Dentistry, Queen Mary University of London, London, UK; 4 Human Development and Health, Faculty of Medicine, University of Southampton, Southampton, UK; 5 Department of Haematology, Royal Bournemouth Hospital, Bournemouth, UK; 6 Division of Hematology, Department of Internal Medicine, The Ohio State University, Columbus, USA

## Abstract

The biological features of *IGHV-M* chronic lymphocytic leukemia responsible for disease progression are still poorly understood. We undertook a longitudinal study close to diagnosis, pre-treatment and post relapse in 13 patients presenting with cMBL or Stage A disease and good-risk biomarkers (*IGHV-M* genes, no del(17p) or del(11q) and low CD38 expression) who nevertheless developed progressive disease, of whom 10 have required therapy. Using cytogenetics, fluorescence *in situ* hybridisation, genome-wide DNA methylation and copy number analysis together with whole exome, targeted deep- and Sanger sequencing at diagnosis, we identified mutations in established chronic lymphocytic leukemia driver genes in nine patients (69%), non-coding mutations (*PAX5* enhancer region) in three patients and genomic complexity in two patients. Branching evolutionary trajectories predominated (*n*=9/13), revealing intra-tumoural epi- and genetic heterogeneity and sub-clonal competition before therapy. Of the patients subsequently requiring treatment, two had sub-clonal *TP53* mutations that would not be detected by standard methodologies, three qualified for the very-low-risk category defined by integrated mutational and cytogenetic analysis and yet had established or putative driver mutations and one patient developed progressive, therapy-refractory disease associated with the emergence of an *IGHV-U* clone. These data suggest that extended genomic and immunogenetic screening may have clinical utility in patients with apparent good-risk disease.

## Introduction

Clinical heterogeneity within chronic lymphocytic leukemia (CLL), especially in the majority of patients presenting with a low-tumour burden, provides a continuing impetus for the discovery of prognostic biomarkers.

Immunogenetic features such as *IGHV* mutation status and stereotypy, immunophenotypic markers, genomic abnormalities and serum markers have prognostic significance. A recently described prognostic index incorporating gender, age, performance status, *IGHV* mutation status, deletions of 11q and 17p, serum B2 microglobulin and thymidine kinase distinguished four risk categories with differing 5-year overall and progression-free survivals.^1^

Candidate gene approaches and next generation sequencing have led to the discovery of mutations in many genes, including *TP53, ATM, NOTCH1, SF3B1, BIRC3, SAMHD1 and EGR2,* with prognostic and/or predictive significance, even when first detected as small sub-clones in the case of *TP53* mutation.^2, 3, 4, 5, 6, 7, 8, 9, 10^ A recent whole-genome study demonstrated the adverse prognostic significance of multiple driver mutations and implicated novel non-coding mutation.^11^ Mutations in an intergenic region on 9p13 correlated with reduced *PAX5* expression and three prime untranslated region (3′UTR) *NOTCH1* mutations associated with a poor outcome comparable to cases with an exon 34 *NOTCH1* mutation. Retrospective analyses of non-trial cohorts show that integration of a restricted set of mutations with copy number data refines and enhances the prognostic significance of the latter and suggest that mutations may be incorporated into future prognostic indices.^12^ Furthermore, copy number array and next generation sequencing data inferred from a single time-point or from sequential studies have demonstrated intra-clonal heterogeneity in CLL, the prognostic significance of sub-clonal mutations and the selective pressure of therapy in determining clonal evolution.^13, 14^ Recent epigenetic data has identified three CLL subtypes that correlate with B-cell maturity and possess distinct patterns of somatic instability, degree of *IGHV* mutation, mutation risk profiles and clinical outcomes.^15, 16, 17, 18^ Despite this progress there remain patients who would be classified as 'low-risk' based on biomarkers who nevertheless have progressive disease.

To obtain more information about the genomic and epigenomic landscape and clinical significance of abnormalities in *IGHV-M* CLL (M-CLL), we performed a longitudinal study at or close to diagnosis, pre-treatment and post relapse in 13 patients. These patients presented with Binet Stage A disease (*n*=10) or clinical monoclonal B-cell lymphocytosis (cMBL) with good-risk biomarkers (*IGHV-M* genes, no del(17p) or del(11q) and low CD38 expression), 10 of whom subsequently required treatment. Using a combination of DNA methylation, copy number analysis, whole-exomic sequencing (WES), targeted deep sequencing (TDR) of recurrently mutated CLL driver genes, screening of non-coding mutation and immunogenetic analysis we identified the presence or acquisition of clonal or sub-clonal driver mutations and DNA methylation changes in eight cases and the emergence of a new immunogenetic clone in one case.

## Methods

### Patient data, copy number and methylation analysis

We studied 13 patients diagnosed at the Royal Bournemouth Hospital between 1992–2007 as cMBL or Binet Stage A, Rai stage 0 CLL according to the 2008 IWCLL/NCI guidelines.^19^ This study was approved by the local Research Ethics Committee and informed consent obtained according to the Declaration of Helsinki. *IGHV* sequencing, CD38, cytogenetic and fluorescence *in situ* hybridisation (FISH) analyses were performed as described^20, 21, 22, 23^ and only cases with mutated *IGHV* genes (excluding major stereotypes), low CD38 expression and lacking 11q or 17p deletion and the availability of stored material were included. Germline DNA (GL) was obtained from saliva (DNAgenotek). CD19+ B-cells were taken at a median of 1 year (0–7.3 years) from diagnosis when patients had cMBL or Stage A disease (time-point 1 (TP1)). The three patients (pts 1–3) who did not require treatment remained as Stage A with a rising (*n*=2) or stable lymphocyte count. All three were sampled again (TP2) at a median of 7 years^6, 7, 8, 9, 10^ from TP1 and one was sampled at further TP (TP3) 3 years after TP2. In the 10 patients requiring treatment, a further sample was taken at a median of 4 months, (range: 0–42) pre-treatment (TP2). In all, 6/10 patients who relapsed after first-line treatment had a sample taken post relapse (TP3) and 2/6 patients were also sampled at relapse following subsequent treatments (TP4 and 5). For 13 sample-trios (GL, TP1 and TP2), DNA regions of copy number alteration and differential methylation were identified using SNP6 arrays (Affymetrix, Santa Clara, CA USA) and 450 K arrays (Illumina Inc., San Diego, CA, USA), respectively, as described.^7, 24^

### Sequencing

WES libraries were prepared from 13 sample-trios (GL, TP1 and TP2) as described.^25^ TDR used Haloplex (Agilent Technologies, Santa Clara, CA, USA) as described^26^ to capture single nucleotide variants (SNVs) identified by WES and 22 genes (exons and 5′ & 3′-UTRs) that are frequently mutated in CLL (Supplementary Table S1) in all tumour samples. TDR libraries were sequenced at high depth (average x4000) to detect mutation down to the 1% level. For each mutation detected by TDR, variant allele frequencies (VAFs) were adjusted for tumour purity estimated as %CD19+ cells. Clonal or sub-clonal mutations were further classified according to ref. 26. We subjected the TDR data to SciClone analysis^27^ to define the clonal dynamics of mutation clusters into three types: (1) static: clusters remain the same over time. (2) Expanding: all mutations in a cluster increase over time. (3) Evolving: new mutations in later samples or one or more mutations in a cluster increase over time. Phylosub was used for tumour phylogeny analysis to predict the most likely order of mutation events and classify either linear or branching evolution patterns.^28^ *PAX5* enhancer region was screened as described in ref. 11. We defined mutated genes into those recurrently mutated from previous CLL studies, non-coding mutation and genes mutated in other haematological malignancies (All excluding copy number changes). Supplementary Methods are available on-line.

## Results

### Genomic landscape of progressive M-CLL

Clinical features, treatment regimens and the genomic landscape at multiple TPs are summarised in Table 1 and Figure 2.

**Figure 1:**
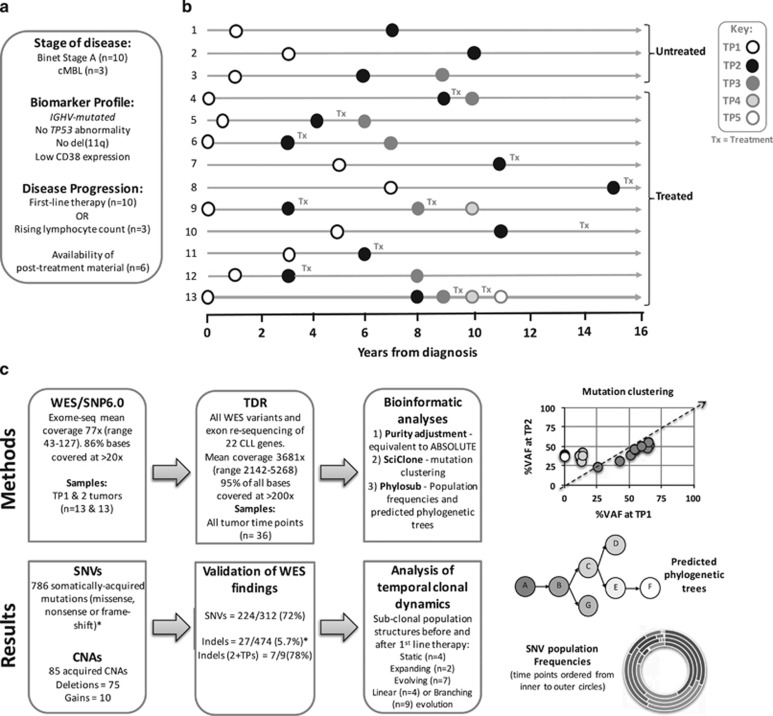
Study overview. (**a**) Inclusion criteria for study and definition of disease progression. (**b**) Tumour TP sampling time line for the 13 patients. Tx, treatment. (**c**) Flow diagram describing genomic analyses and result summaries. Example data plots for SciClone mutation clustering, Phylosub phylogenetic trees and concentric pie charts (each layer, inner to outer, is a sampling TP) displaying imputed SNV population frequencies at each phylogenetic node. *For indel filtering we accepted a high-false positive WES rate to ensure we could capture all of the 'true' somatically acquired indel variants by TDR (Supplementary Methods). When considering indels present in two or more tumour TPs (2+TPs) our indel TDR validation rate (78%, 7/9%) was in line with the SNV rate (72%).

We employed WES and TDR to identify somatically acquired mutation in tumour samples from 13 cases with mean coverage of 77x (min–max: 43–127) and 3681x (2142–5268), with >86% of all bases covered at >20x and >200x, respectively. (Figure 1, Supplementary Tables S2 and S3). Of the filtered WES variants (Supplementary Table S4), TDR confirmed the presence of 224/312 (72%) SNVs and 7/9 (78%) indels (when present at both TP1 and TP2), respectively (Supplementary Table S5 and Supplementary Methods). We used the TDR variants to study temporal clonal evolution and demonstrated that our TP1 and TP2 samples harboured a similar mutation burden, with on average 17 (min–max: 9–26) and 19,^8, 9, 10, 11, 12, 13, 14, 15, 16, 17, 18, 19, 20, 21, 22, 23, 24, 25, 26, 27, 28, 29^ respectively. After adjusting for tumour purity, we observed no difference in the mean number of clonal (6 vs 6) or sub-clonal mutations (10 vs 12) in either untreated TP (TP1 vs TP2). All reported VAFs (%VAF) are adjusted for tumour purity.

**Figure 2:**
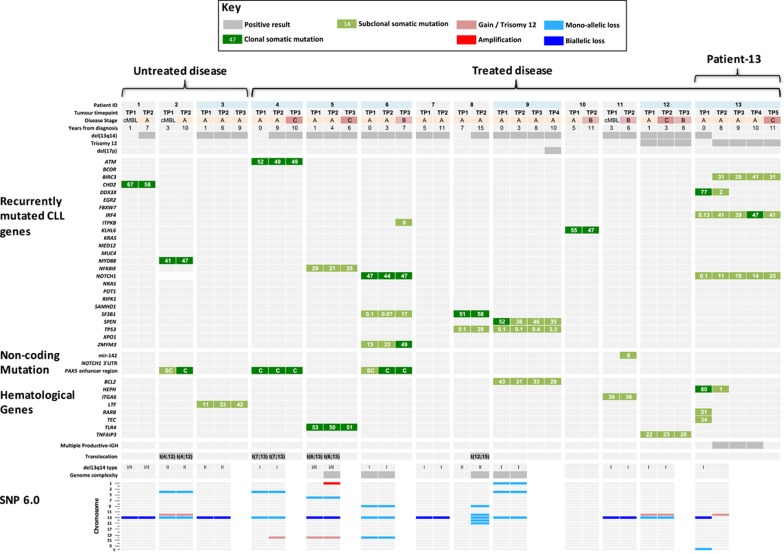
Heat-map representation of tumour TPs analysed by WES and targeted deep re-sequencing. From top to bottom: key to heat-map cell shading. Patient characteristics, light blue cell shading indicates patients with follow-on tumour samples (that is, TP3, TP4 and TP5); dark-grey cells indicated a positive result. Sub-clonal (light-green cells) and clonal mutations (dark-green cells) in each patient, grouped into recurrently mutated CLL driver genes, non-coding mutation described in Puente *et al.*^6^ and genes mutated in haematological malignancies. Numbers in cells denote tumour purity-adjusted %VAFs from TDR. SC, sub-clonal; C, clonal from Sanger-seq traces. Presence of multiple productive-*IGH* relating to patient 13, chromosomal translocation or genome complexity is denoted by dark-grey cells. SNP6.0 data for TP1 and TP2 samples.

Focusing on genes previously shown to be recurrently mutated in CLL; at TP2, clonal mutations were detected in *MYD88* (p.L265P; pt-2), and *CHD2* (pt-1) among the three untreated patients and in *ATM* (pt-4), *DDX3X* (pt-13), *NOTCH1* (pts-6,13), *SF3B1* (pts-6, 8), *TP53* (pts-8, 9), *NFKBIE* (pt-5), *SPEN* (pt-9), *ZMYM3* (pt-6), *KLHL6* (pt-10), *BIRC3* (pt-13) and *IRF4* (pt-13) among the 10 patients who received treatment. Only five of these mutations were clonal (Figure 2). In addition, three patients (pts-3, 11, 12) exhibited missense mutations (damaging by Polyphen-2) in genes known to have a role in other haematological tumours, *LTF* (pt-3), *ITGA6* (pt-11) and a frame-shift in *TNFAIP3* (pt-12) and all were present at sub-clonal levels (11–42% VAF). Only one case (pt-7) lacked any recurrently mutated driver mutation documented in CLL or other haematological malignancies. However, WES did identify 10 mutated genes from which two candidates emerged, namely missense mutations in *ZBTB7C* a kidney cancer-related gene that interacts with p53 (ref. 29 and *S1PR4* a receptor expressed in hematopoietic cells that interacts with *MAPK3* (ERK1), placing it in the B-cell receptor pathway.^30^

The CLL driver mutations (with the exception of *BIRC3* in pt-13) were detectable (by the presence of one or more mutated reads) at TP1 supporting the hypothesis that identification of mutations at diagnosis may identify individuals later requiring therapy. The CLL driver gene mutations with VAFs <1% (*IRF4*, *NOTCH1, SF3B1 and TP53;* in pts-6, 8, 9, 13) at TP1 were ascertained by manual curation of the TDR sequencing reads (Supplementary Table S6) after being originally detected in later tumour TPs with a higher VAF, suggesting a larger sub-clonal population at progression. Pileup of reads across all samples provided statistical confidence for calling *TP53* mutation in patient 8 and 9 below a 1% VAF (*P*=0.013 & *P*<0.001; Supplementary Table S6). Droplet-digital PCR analysis of patient 13 confirmed the presence of the *NOTCH1* mutation at TP1 (Supplementary Figure S1). Together, this would suggest sequencing depths much >x4000 will be required to robustly identify all sub-clonal mutations, for example, patient 8 had a *TP53* mutation (29%VAF) at TP2, detectable at TP1 in 9/15581 reads (0.1%VAF), equating to the presence of one mutant cell in ~1000 CLL B-cells. In patient 13, the *BIRC3* mutation at TP2 was not identified at TP1 (0/3624 reads) and conversely a clonal *DDX3X* mutation at TP1 was detected as a small sub-clone at TP2. This lead to a re-appraisal of this case which is discussed in detail later. At relapse, we identified mutations in *SF3B1* (pt-6) and *TP53* (pt-9) with VAF's of 17 and 3.3%, respectively, which had VAF's of <1% pre-treatment.

We screened for non-coding mutations.^11^ *PAX5* enhancer region mutations were detected in three patients, estimated at TP1 to be clonal in one case (pt-4) and sub-clonal in the other cases (pts-2, 6; Figure 2 and Supplementary Figure S2). These mutations co-occurred with other mutations: *MYD88* (pt-2), *ATM* (pt-4) and *NOTCH1, SF3B1* and *ZMYM3* (pt-6). We also detected at TP2, the presence of a sub-clonal mutation (6% VAF; chr17:56408615:T>C) in the mature sequence of hsa-mir-142, this co-occurred with a *ITGA6* mutation in patient 11 (Supplementary Figure S2). The *NOTCH1* 3′UTR mutations, previously observed solely in cases of U-CLL,^11^ were absent.

Combining karyotypic, FISH and SNP6 data, at TP1, only two patients (pts-8, 10) had no copy number abnormality or translocation, while the remainder had mono (*n*=5) or mono+biallelic loss of 13q14. Two patients (pts-12, 13) with del13q also had trisomy 12. Two patients (pts-6, 9) had a complex genome (⩾3 copy number alterations), defined as previously reported.^31^ SNP6 confirmed the absence of 11q or 17p deletion (Supplementary Table S7). At TP2, additional abnormalities were detected in three patients (pts-4, 5, 8). Interestingly, the complex karyotypic abnormality in patient 8 was associated with expansion of a sub-clonal *TP53* mutation (from 0.1% at TP1 to 29% at TP2) without *TP53* loss. Three patients (pts-2, 4, 5) had an unbalanced translocation. Patient 13 had a remarkable change in copy number and is discussed later.

### Intra-clonal heterogeneity in progressive M-CLL

Our longitudinal approach provided an opportunity to evaluate intra-clonal heterogeneity both before and following therapy.

SciClone analysis of TP1 and TP2 data from patients before therapy enabled us to make the following observations: (1) Four cases (pts-5, 7, 10, 12) had a static sub-clonal structure with mutation clusters present at similar VAFs at both TPs (pt-12 in Supplementary Figure S3A). (2) Two cases (pts-2, 4) had an expanding population where all mutations in a cluster were more dominant at TP2 (pt-4 in Supplementary Figure S3A). (3) Seven patients (pts-1, 3, 6, 8, 9, 11, 13) had an evolving genome where new mutations appeared (*n*=8 in four patients (pts-6, 8, 9, 13) and/or one or more mutations in a cluster increased, at TP2 (pt-6 in Supplementary Figure S3A). Six of these new mutations had low read depths (< × 4000; ranging: 138–3642) in TP1 samples, suggesting there may be a lack of detection sensitivity. The three remaining mutations (*DSG4*, *SIM1* and *SLC8A2*) had adequate depth (4218–9043) at TP1, suggesting these mutations are either very rare (in <0.5–1% of cells) or represent acquired mutations at disease progression (TP2). For the six patients with post therapy TPs, there were no new mutations and we observed two patients (pts-5, 12) exhibiting a static structure with same distribution of sub-clones pre- and post-treatment (pt-12 in Supplementary Figure S3B), while four cases (pts-4, 6, 9, 13) showed expansion (pt-4 in Supplementary Figure S3B) or evolution (pt-6 in Supplementary Figure S3B) following an apparent therapy-related sweep selecting resistant/fitter sub-clones. Interestingly, these four cases had either expanding or evolving mutation clusters before therapy. Results for the remaining patients are provided in Supplementary Figure S4.

Phylosub analysis predicted a linear evolutionary path, where progeny replaced ancestral clones, in a minority of patients (*n*=4) in whom SciClone analysis had identified either static (pts-7, 12) or expanding (pts-2, 4) mutation clusters. Phylosub also predicted that the *ATM*, *MYD88, S1PR4, TNFAIP3* and *ZBTB7C* mutations in these patients were early evolutionary events (placed in the first or second nodes of each tree). Complex branching trajectories were predicted in the remaining nine patients, including two of the three patients (pts-1, 3) with no indication for therapy, and provided the following insights: (1) The *BCL2*, *CHD2*, *NOTCH1*, *SF3B1* and *TLR4* mutations were all predicted to be early events. (2) Generally located at branch points, the sub-clones that appear to have good fitness, or are selected for at later tumour TPs, contained CLL drivers (*ITPKB*, *NFKBIE*, *SF3B1*, *TP53* and *ZMYM3*) or genes mutated in other haematoloical malignancies (*ITGA6 and* *LTF*) supporting the role of genes in the latter two categories as candidate drivers of progression in those patients. (3) Convergent evolution was only found in two patients (pts-4, 8) who exhibited two mutations in a single gene (*ATM*:p.I2606M/p.Q2733K and *IGLL5:*p.C31Y/p.P50S). All four mutations were clonal and only the *IGLL5* mutations were close enough to be detected on separate overlapping reads, but as the VAFs were similar (54–57%) they could have arisen in the same cell.

**Figure 3:**
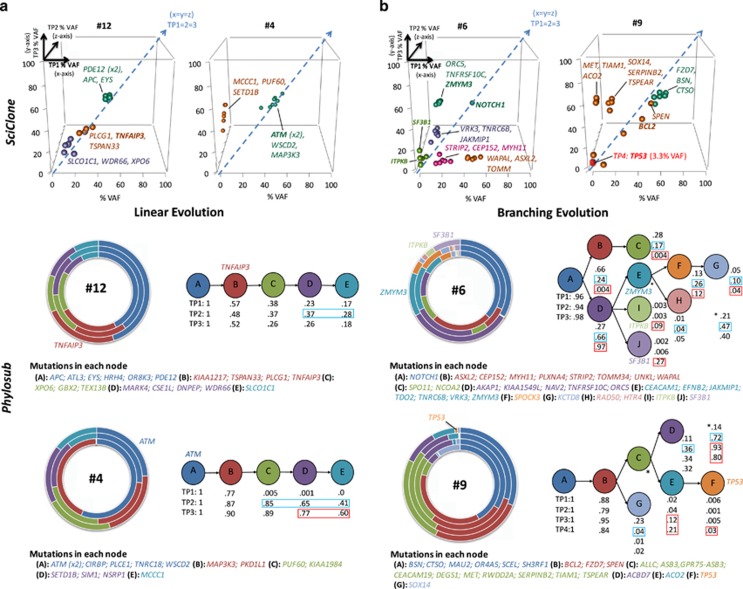
PhyloSub analysis of TDR results in predicted linear and branching clonal evolutionary pattern. By columns (**a**) two patient examples (pts-12 and 4) of linear evolution path, (**b**) two patient examples (pts-6 and 9) of complex branching trajectories. (**a** and **b**) Top panel: XYZ scatter-graphs displaying the SciClone mutation clustering analysis on TDR data sets from sequential tumour TPs TP1 (*x* axis; first tumour sample), 2 (*z* axis; progression) and 3 (*y* axis; post-treatment). Data point symbols denote a distinct mutation cluster and the *x*=*y*=*z* line is displayed as a dashed blue arrow and denotes no change in the tumour purity-adjusted %VAF of mutation clusters between TPs (clonal equilibrium). Selected gene symbols are displayed adjacent to its corresponding mutation cluster. (**a** and **b**) Bottom panel: concentric pie charts (each layer, inner to outer, is an early to later sampling TP) displaying imputed SNV population frequencies at each phylogenetic node. Predicted phylogenetic tree structure (best model shown), with population frequencies for each node from Phylosub analysis. Blue and red boxes denote large changes SNV population frequencies before and after first-line treatment, suggesting ongoing clonal dynamics and selection by therapy, respectively. Selected gene symbols are displayed adjacent to the corresponding segment of the pie chart or phylogenetic node.

Phylosub and SciClone analysis of patients-4, 6, 9 and 12 is displayed in Figure 3. These four patients had three (pts-4, 6, 12) or four (pt-9) tumour TPs presenting static, expanding or evolving mutation clusters with predicted linear (pts-4, 12) or branching (pts-6, 9) evolution trajectories before and after therapy. At TP2 in patient 4 we observe expanding populations (nodes C–E) replacing the ancestral population, suggesting that these later mutations are associated with disease progression. Following treatment with bendamustine plus rituximab (TP3), we observed no reduction in population frequencies. Conversely, in patient 12, we observed a reduction back to baseline (TP1) for nodes C–E, suggesting a similar sensitivity of the descendant clones to chlorambucil–rituximab therapy; this patient remains in remission. Interestingly, in patient 6 the *ITPKB*, *SF3B1* and *ZMYM3* mutations were predicted to be in distinct populations (nodes I, J and E, respectively) and following chlorambucil treatment at TP3 the population frequencies of both the I (*ITPKB*) and J (*SF3B1*) nodes increased in comparison to other nodes. In patient 9 following two rounds of therapy (chlorambucil and bendamustine plus rituximab) we observed 75% del(17p) loss by FISH and a sub-clonal *TP53* mutation (p.Y234C, 3.3%VAF), at TP4. A complex karyotype was also observed at TP3. The difference between del(17p) FISH clone size and %VAF of the *TP53* mutation would suggest evolutionary independent events, with a rare *TP53* mutated clone detectable at presentation and later acquisition of del(17p) loss in another *TP53*-wildtype clone. Following exposure to chemo-immunotherapy, the resistant *TP53* aberrant clones accumulate and dominate the tumour. Phylosub results for the remaining patients are provided in Supplementary Figure S4.

### The emergence of an *IGHV-U* immunogenetic clone can drive progression

**Figure 4:**
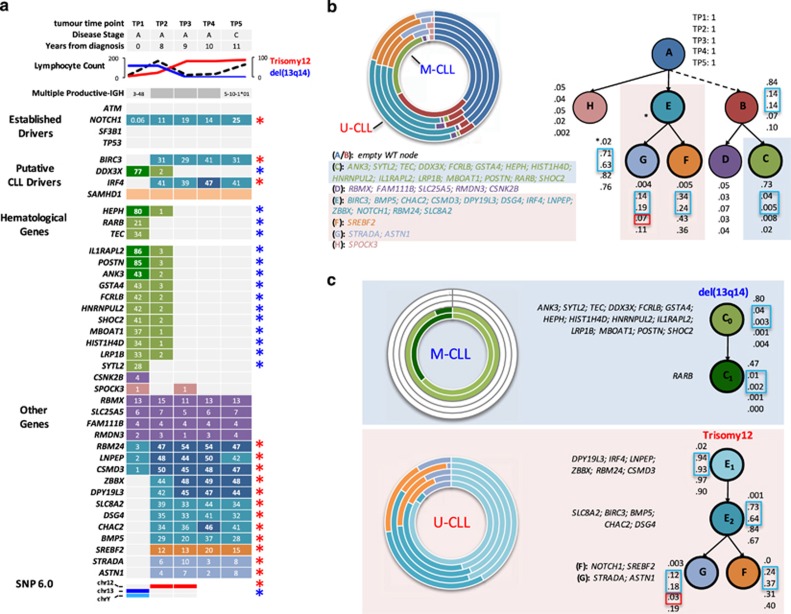
Evolution of multiple productive-*IGH* in CLL patient 13. (**a**) From top to bottom: five tumour TPs with corresponding clinical, cytogenetic and immunogenetic data. Mutation heat-map representation of five tumour TPs analysed by targeted deep re-sequencing. Numbers in cells denote tumour purity-adjusted %VAFs from TDR. Cell colours are linked to the SNV population nodes/frequencies displayed in part **b**. Lighter shading indicates a sub-clonal mutation. Blue asterisks/del13q14=M-CLL clone (IGHV3–48; 92% identity to germ-line; del(13q14)) and red asterisks/trisomy 12=U-CLL clone (IGHV5–10*01; 100% identity to germ-line; trisomy 12). (**b** and **c**) Filled light blue and red boxes denote mutations and cytogenetic abnormalities inferred into the M-CLL and U-CLL clone, respectively. From left to right: concentric pie charts (each layer, inner to outer, is an early to later sampling TP) displaying imputed SNV population frequencies at each phylogenetic node. Predicted phylogenetic tree structure, with population frequencies for each node from Phylosub analysis. Best models are displayed for analyses using all mutations (**b**) or only mutations associated with either the M-CLL or U-CLL clone providing insights into the probable order of mutation (**c**). Note from TP3 onwards the mutations associated with the M-CLL clone are not detectable by sequencing. Open blue and red boxes denote large changes SNV population frequencies before and after first-line treatment, suggesting ongoing clonal dynamics and selection by therapy, respectively.

Substantial differences in the clone size of copy number alterations and mutations between TP1 (diagnosis) and TP2 (+8 years) in patient 13, and the detection of a *BIRC3* mutation only at TP2, led to a review of karyotypic, FISH, SNP6 and mutational data and targeted re-sequencing analysis of samples taken after treatment with chlorambucil, bendamustine plus rituximab and ofatumumab, all of which were ineffective. This showed a remarkable temporal shift in genomic aberrations supporting a dominant population at diagnosis containing a deletion of 13q, loss of chromosome Y and a unique set of mutations, including *DDX3X, HEPH, RARB* and *TEC.* These were gradually replaced by a 47, XY, trisomy 12, population with mutations in 18 genes including *BIRC3*, *NOTCH1* and *IRF4* (Figure 4 and Supplementary Table S8). As the dominant mutations present at TP2 are more frequently or exclusively associated with *IGHV-U* genes, we reanalyzed the *IGHV* status at TP2, and identified a dominant IGHV5-10-1*01 (100% identity to germ-line) clone in addition to the *IGHV3–48* clone with 92% germ-line identity present at diagnosis. *IGHV* analysis of six intermediate samples between TP1 and 2 detected the *IGHV-U* clone as far back as 4 years post diagnosis (Supplementary Table S9). Importantly, both the *NOTCH1* mutated and trisomy 12 sub-clones were detectable at diagnosis using TDR and FISH, respectively, demonstrating the presence of the IGHV5-10-1*01 clone at diagnosis but at a level which was undetectable using standard immunogenetic assays. This patient's tumour eventually transformed to a diffuse large B-cell lymphoma, using the IGHV5-10-1*01 clone. Unfortunately, TDR of the Richter's node biopsy was not successful.

Additional *IGHV* sequencing on the other cases failed to identify any additional patients with evolution of an *IGHV-U* clone (Supplementary Table S9).

### DNA methylation subtyping and co-evolution of epigenetic changes

We performed clustering analysis of the TP1 methylation data together with a reference sample set where the three epigenetic subtypes were defined previously (Oakes, in press)^32^ and determined that 12/13 patients belonged to the high-programmed CLL DNA methylation subtype, consistent with the selection of our patients based on the presence of mutated *IGHV* (Supplementary Figure S5A). In all except a single patient, adjacent clustering of the TP2 data confirmed the clonal relationship between tumour TPs. The exception was patient 13 which clustered in the low-programmed CLL subtype at TP2, consistent with the emergence of the *IGHV-U* clone. Patients with limited genetic evolution had relatively few differences in overall methylation, where as those that exhibited either expansion or evolution of genetic sub-clones showed higher proportions of altered CpG methylation (Supplementary Figure S5B).

## Discussion

Patients with *IGHV-M* genes, defined as <98% identity to the germ-line sequence, have a better outcome than those with *IGHV-U* genes.^33, 34^ While stable cMBL and Stage A CLL are strongly enriched for cases with M-CLL, 37–39% of patients entered into the UKCLL4 and CLL8 trials of first-line therapy had M-CLL^8, 35^ and the key biological features responsible for progression are still poorly understood.^11^ M-CLL is biologically heterogeneous and studies have shown that CD38, CD49d and ZAP70 expression, serum markers, stereotypic subset-2,^36^ telomere length, del(11q) and del(17p) and genomic abnormalities influence time to first treatment or outcome following therapy.^37, 38, 39, 40, 41, 42^ In this study, we performed longitudinal genomic and epigenomic characterisation before and after therapy in a cohort of M-CLL cases presenting with Stage A disease or cMBL. All cases lacked established biomarkers associated with progression: namely, high CD38 expression, del(11q), del(17p). Nevertheless, 10/13 cases subsequently required treatment.

Before treatment we found mutations in genes that are recurrently mutated in CLL, (*ATM*, *BIRC3*, *CHD2*, *DDX3X*, *IRF4*, *ITPKB*, *KLHL6*, *MYD88, NOTCH1*, *NFKBIE, SF3B1*, *SPEN*, *TP53 and ZMYM3*) in nine patients (69%) with a mean of one mutation per case (min–max: 0–4). This mutation incidence is consistent with a recent whole genomic and exomic study, where 83% of M-CLL cases had a driver mutation.^11^ Nine patients had one or more clonal mutations detected pre-treatment and the majority of the sub-clonal mutations were detectable at the earliest TP at or soon after diagnosis. Although many of the above genes are associated with disease progression and/or resistance to treatment, the clinical significance of others (*ITPKB, KLHL6 and SPEN*) is less certain. A further three patients had mutations in other genes (*LTF*, *ITGA6* and *TNFAIP3*) implicated in other haematological malignancies (COSMIC v 73;^43^).

We also screened for non-coding mutation in the *PAX5* enhancer region. These mutations co-existed with *MYD88*, *ATM, NOTCH1*, *SF3B1* and *ZMYM3* mutations in contrast to the original description where they were either the sole recurrent mutation or occurred in conjunction with 13q loss.^11^

SciClone and Phylosub analysis provided novel insight into the extent of intra-clonal heterogeneity. Clonal expansion or evolution, predominantly in a branching pattern, was found in nine cases pre-treatment, indicating that sub-clonal competition occurs in the absence of selective pressure through therapy, with the resistant/fitter sub-clones dominating the tumour at post therapy TPs. A recent WES study comparing matched pre-treatment and relapse samples demonstrated that clonal evolution was the rule after therapy and the resistant clone could be detected before treatment in ~30% of cases.^14^ Six cases developed isolated splenomegaly, two had splenomegaly and lymphadenopathy and two had lymphadenopathy before treatment. Further spatial-temporal studies will be required to determine the site(s) of clonal evolution. Phylosub analysis also demonstrated the selection of sub-clones containing either *SF3B1*, *TP53, ITGA6*, *ITPKB*, *LTF*, *NFKBIE*, or *ZMYM3* mutations, supporting their role as candidate drivers of progression.

One unexpected finding was the emergence of an *IGHV-U* clone in one patient who after 6 years of stable disease, developed progressive, therapy-refractory disease and culminating in a clonally related lymphomatous transformation. Each clone had a unique spectrum of gene mutations and epigenetic profiles consistent with two distinct and competing leukaemic clones originating from a different pool of lymphocyte progenitors. Bi, or more rarely multi-clonal *IGHV* rearrangements have been documented. From a cohort of 1147 cases, Plevova *et al.* identified seven cases with both mutated and unmutated clones in which serial studies showed diminution of an *IGHV-M* clone with persistence of a co-existing *IGHV-U* clone, resulting in re-classification to U-CLL.^44^ Clinically, this was associated with progressive lymphocytosis, disease progression and in some cases, the selection of a *TP53*-defect post therapy. Our case is unusual in that the unmutated clone was not detectable until 4 years after diagnosis using standard methodologies for *IGHV* sequencing, even though a sub-clonal trisomy 12 and *NOTCH1* mutation, associated with the unmutated clone were detected at diagnosis using more sensitive techniques.

Previous whole-genome longitudinal studies of copy number^45, 46, 47^ and/or genomic mutations^48, 49, 50, 51^ in CLL have included a higher percentage of cases with U-CLL than M-CLL and with progressive rather than stable disease. However, a picture has emerged of clonal evolution which is usually branched rather than linear and is more frequent in cases with progressive disease who have required treatment for which the majority of driver mutations can be detected at initial testing, often as small sub-clones. Our study confirms many of these findings but also highlights the high frequency of CLL driver mutations in a progressive cohort miss-labelled as 'good-risk' and the extent of clonal evolution before therapy. DNA methylation analysis revealed that co-evolution of genetic and epigenetic changes is a prominent feature and that this exists regardless of *IGHV* subtype and mutational risk assessment, supporting the perspective that evolution is an important predictor of disease progression.^13, 14^ A recent study^52^ also found the highest number of differentially methylated CpGs were in cases with genetically evolving and expanding sub-clones.

From a clinical perspective, a key question is whether the additional information that genomic and epigenomic screening provides in this group is likely to improve patient outcome. Although it would be unwise to draw general conclusions from this small study, it does offer three examples where screening could have clinical utility. Firstly, 2/13 cases had small *TP53* mutated clones early in the disease with evidence of clonal selection post therapy. These cases had no *TP53* loss detectable by FISH when the mutant clone was initially detected. Secondly, 6/11 cases with 13q loss fell into the 'very-low' risk category defined by Rossi *et al.* in which predominantly *IGHV-M* cases with isolated 13q loss, lacking mutations in *TP53, BIRC3, NOTCH1, SF3B1* and *MYD88* had a prolonged time to first treatment and an expected overall survival similar to the matched general population.^12^ Two of our six cases with isolated 13q loss had a progressive lymphocytosis with no indication for treatment and a mutation in *CHD2* or *LTF* while three of the four who required treatment had mutations in *NFKBIE, TNFAIP3* or *ITGA6* suggesting that more extensive screening may aid in the differentiation of cases destined to have stable or progressive disease. Finally, the emergence of an *IGHV-U* clone, not evident at diagnosis, which eventually lead to the patient's death is a rare event but supports either repeat immunogenetic analysis in cases with unexplained progressive or therapy-refractory disease, or the use of more sensitive assays capable of detecting small clones. In summary this study does not evaluate the role of other factors such as cell signalling as an explanation for disease progression, but does support the role for sequential genomic/epigenomic screening as a means of identifying potential driver mutations and predicting progressive disease.

## Figures and Tables

**Table 1:** Overview of patient biomarker and clinical data

*Patient ID*	*Age at diagnosis*	*Tumour time-point 1*				*Tumour time-point 2*				*LDT*	*First treatment (response)*	*Treatment at relapse (response)*	*Current status at last follow-up (03/8/15)*
		*IGHV (% identity)*	*FISH/karyo*	*%CD38 expression*	*Lymphocyte count (x10^9/L)*	*IGHV (% identity)*	*FISH/karyo*	*%CD38 expression*	*Lymphocyte count (x10^9/L)*				
1	52	IGHV4–61 (93)	del(13q) +/− (54%)	2	6	IGHV4–61 (93)	del(13q) +/−,−/− (10, 85%)	1	142	> 1year	–	–	Stable CLL
2	72	IGHV3–73 (91)	del(13q) 46, XY, der4(4)t(4;12)(q35;q13)	1	5	IGHV3–73 (91)	No change	1	25	> 1year	–	–	Stable CLL
3	57	IGHV2–70 (93)	del(13q) +/− (90%)	1	92	IGHV2–70 (93)	No change	1	88	> 1year	–	–	Stable CLL
4	61	IGHV4–59 (89)	del(13q) +/− (6%) 45, X-Y, t(7,13)(a11.2;q14)	15	34	IGHV4–59 (89)	del(13q) +/−,−/− (76, 8%), 45, X –Y, t(7;13)(q11.2;q14)	50	136	> 1year	BR (CR)	–	High-risk MDS. Died.
5	79	IGHV4–61 (92)	del(13q) +/−,−/− (9/86%) 46, XY, t(6;13)(q26;q14)	1	107	IGHV4–61 (92)	del(13q) +/−,−/− (14/86%) 46, XY, t(6;13)(q26;q14)	–	198	> 1year	Chlor (CR)	BR (PR)	Stable CLL
6	70	IGHV3–48 (97)	del(13q) +/− (88%)	1	20	IGHV3–48 (97)	del(13q) +/−,−/− (53, 10%)	2	127	6–12 months	Chlor (GR)	BR (CR)	In remission
7	47	IGHV4–34 (92)	del(13q) +/−,−/− (19, 72%)	6	59	IGHV4–34 (92)	No change	1	81	> 1year	Chlor R (PR)	Alemtuz (CR, MRD +ve)	In remission
8	59	IGHV3–23 (96)	Normal	3	21	IGHV3–23 (96)	del(13q) +/− (66%) 46, XY, del(9)(q21), t(12;15)(p11;q15)	1	48	> 1year	Chlor Of (CR)	–	In remission
9	74	IGHV3–7 (89)	del(13q) +/− (54%)	1	17	IGHV3–7 (89)	del(13q) +/− (91%) (+ del17p at TP3)	1	185	6–12 months	Chlor (PR)	BR (CR)	On Ibrutinib
10	56	IGHV3–48 (93)	Normal	5	181	IGHV3–48 (93)	No change	2	139	> 1year	Chlor (PR)	Continuum	Stable CLL
11	64	IGHV3–23 (91)	46, XY. No 13q FISH	1	58	IGHV3–23 (91)	NT	1	145	> 1year	B Of (CR)	–	In remission
12	63	IGHV4–34 (96)	47, XY,+12. No 13q FISH	1	42	IGHV4–34 (96)	NT	1	77	6–12 months	Chlor R (CR)	BR (CR, MRD +ve) Continuum	In remission
13	67	IGHV3–48 (92)	Tri 12 (2%) del(13q) −/− (55%)	9	18	IGHV3–48 (92) & IGHV5-10-1*01 (100)	Tri 12 (73%) (Tri 12 (75%) at TP3)	21	158	> 1year	Chlor (NR)	BR (PR), Of (PR)	Richters syndrome, NR to CHOP of. Died.

Abbreviations: BR, bendamustine plus rituximab; FISH, fluorescence *in situ* hybridisation; LDT, lymphocyte doubling time (from diagnosis for the first year of follow-up); NT, not tested.
