# Supplementary material for: Longitudinal copy number, whole exome and targeted deep sequencing of 'good risk' IGHV-mutated CLL patients with progressive disease
Source: Leukemia. 2016 Feb 26;30(6):1301–10. doi: 10.1038/leu.2016.10 (PMC4861248; doi:10.1038/leu.2016.10)
Supplement: Supplementary Information [file leu201610x1.doc]

# ****Supplementary Methods****

**Sample Prep**

Buccal cells (GL) were obtained in all cases using the Oragene DNA kit (DNA Genotek) and tumour cell contamination of the germ-line material was excluded by PCR and heteroduplex PAGE analysis utilizing BIOMED2 protocol FR and J-Hc consensus primers specific for the tumour IGHV re-arrangement (1). DNA was extracted from all samples using standard approaches.

**Genome-wide copy number analysis**

CNA's resulting from unbalanced translocations were not counted as a separate genomic event.

**Whole exome sequencing**

Sequencing libraries were prepared from 13 samples trios (GL, TP1, TP2) for whole exome sequencing (WES) using targeted exome capture (SureSelect Human All Exon Agilent) prior to high-throughput paired-end sequencing with the Illumina HiSeq system and downstream analysis. In brief, somatic single nucleotide variants (SNVs) were identified in tumour and GL sequencing reads, where a minimum variant allele frequency (VAF) threshold of 10% with a minimum read depth of 4 was employed to identify high-confidence variants, prior to additional filtering to remove clusters of false positives and SNV calls near indels with the same frequency and depth thresholds (n=312 SNVs). Our small insertion and deletion (indel) call rate after filtering is higher than expected (444 frameshift deletions and 30 frameshift deletions. 60% of all filtered variants (474/786)), this is part of our study design. For indel calling, we removed the minimum variant allele frequency (VAF) threshold of 10% and set the minimum read depth of alternative reads to 2. We accepted this artificially high-false positive rate (94%, 447/474 filtered WES indels not confirmed by TDR) to ensure we could perform orthogonal capture and identify all of the 'true' somatically-acquired indel variants by the targeted deep re-sequencing experiments (5.7% 27/474 filtered WES indels confirmed by TDR). When only considering indels present in 2 or more tumour time-points our indel validation rate (78%, 7/9%) was in line with the filtered SNV rate (72%, 224/312). Variants were annotated with respect to genes and transcripts and filtered using the Annovar software tool (v2012Jun21) (2). Variants were cross referenced with databases of known variation were downloaded from the Annovar website (June 2012); data from the 1000 Genomes Project (2012 April release) (3), dbSNP135 (and a version with SNPs flagged as rare <1% frequency or clinically associated by NCBI) and data from 4300 European American samples from The National Heart Lung and Blood Institute Exome Sequencing Project Exome Variant Server (http://evs.gs.washington.edu/EVS/), (ESP6500 release). Finally variants were annotated with functional prediction scores, Phylop, SIFT, Polyphen2, lrt, mutationtaster, gerp++, and Grantham scores.

**Haloplex targeted re-sequencing**

The targeted re-sequencing assay was designed using the Haloplex targeted DNA enrichment capture (500kb. Agilent Technologies), to capture SNVs identified by WES and exons of twenty-two genes that are most frequently mutated in CLL (**Supplementary table 1**) in 13 matched TP1 and TP2 samples (**See table 1**) and additional time points after first-line therapy (n=8) using the Illumina HiSeq system at high depth (average x4000 fold) allowing for detection of sub-clonal mutation down to the 1% level. In 225ng genomic DNA from 13 matched TP1 and TP2 samples (**See table 1**) and additional time points after first-line therapy (n=8) using the Illumina HiSeq system at high depth (average x4000 fold). At this sequencing depth sub-clonal mutations can be detected down to the 1% level, assuming a minimum of observation of 4 sequencing reads containing the variant base, a Q40 phred like base quality score (p(detected) = 99.99) and a cumulative binomial distribution for n read depth [
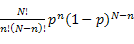
]. Variants were called based on a minimum read depth of 4 and a minimum phred scaled quality score of 20.

**Identification of clonal and sub-clonal mutations**

Clonal mutations, defined as the variant allele frequency (VAF) adjusted for tumour purity equal to or larger than 0.45, were determined by a binomial distribution using the number of alternative allele reads, the total number of reads from cancer cells (approximately, total number of reads × purity estimate), and the expected VAF of 0.45. Mutations with cumulative probability distribution function p < 0.07 were identified as subclonal. The threshold was chosen to have the best performance when compared with ABSOLUTE clonal calls, with the False Discovery Rate (FDR) of 10%, if the ABSOLUTE results were treated as true positives, based on previous 7 SMZL cases with SNP6.0 data (4). The ratio of observed VAF over the value of half of purity estimate was also calculated for individual mutations. This was the same as an ABSOLUTE-derived CCF (Cancer Cell Fraction) when the ratio was less than 1 and the mutation was not coupled with local copy number changes. When the ratio was larger than 1 for mutations with successful binomial tests, it was further adjusted as 1. Those mutations with the unadjusted (raw) ratio much larger than 1 (e.g., >2) were very likely affected by local copy number aberrations.

**SciClone and Phylosub analysis**

SciClone is an approach based on variational Bayesian beta mixture modeling to identify low frequency subclones by clustering the tumour purity-adjusted VAFs (described above) from copy number neutral, LOH-free, non-repetitive regions of the tumour genome. The method automatically infers the optimal number of clusters based on an initial overestimation of their expected number (defaulted to a max of 10 and reduced in 4 in patients with mutation counts <10). Each cluster is represented by a posterior predictive density, which provides the probability of a VAF given the observed data (and subsequent model fit). These densities probabilistically define boundaries between the different clusters. The SciClone package (version 1.0.7) was ran in R (version 1.1.1) and can analyze each tumour time-point individually, or as matched-pairs or trios, generating 1D, 2D and 3D scatter graph representations of mutation clusters enabling visualization of the temporal patterns of CLL tumour evolution in our patients.

Phylosub was utilized to reconstruct the evolutionary history from tumour purity adjusted mutation frequencies across the multiple tumour samples. This Bayesian non-parametric statistical model infers the phylogeny and genotype of major sub-clonal lineages represented in the cancer cells. When multiple phylogenies are consistent with a set of mutation frequencies phylosub represents the uncertainty in tumour phylogeny using mutation partial order plots. We chose to display one of the top three phylogenetic tree outputs ('top_K_trees output'), selecting the tree structure with the fewest empty nodes.

**450K Methylation analysis**

High-quality genomic DNA (500 ng) was bisulfite converted using the EZ DNA Methylation Gold Kit (Zymo Research). The Infinium methylation assay was carried out as described previously (5). Data from the 450k Human Methylation Array were normalized by the Beta Mixture Quantile (BMIQ) method (6) using the RnBeads analysis software package (7). To explore the longitudinal change in genome-wide DNA methylation, 450K datasets were compared between time points in all 13 CLL cases. To assess DNA methylation stability between tumor time-points, all hyper- and hypo-methylation differences of greater than 10% (out of a total of 459,625 CpGs analyzed) were tallied per case and were adjusted for differences in tumor cell content between samples and genomic copy number changes as previously described (8). Our 450K data was also combined with 127 CLL cases from a previously published 450K dataset where the DNA methylation subtypes are known (9) and as previously described (9-11). Co-clustering was performed as done previously (9), using the top 1,000 most variable CpGs across all samples to assign each patient to either the high, intermediate and low programmed CLLs (HP-, IP-, and LP-CLLs, respectively).

**References**

1 van Dongen JJ, Langerak AW, Brüggemann M, Evans PA, Hummel M, Lavender FL, et al. Design and standardization of PCR primers and protocols for detection of clonal immunoglobulin and T-cell receptor gene recombinations in suspect lymphoproliferations: report of the BIOMED-2 Concerted Action BMH4-CT98-3936. Leukemia 2003;17(12):2257-317.

2 Wang K, Li M, Hakonarson H. ANNOVAR: Functional annotation of genetic variants from next-generation sequencing data. Nucleic Acids Research. 2010;38:e164.

3 Consortium. GP, Abecasis GR, Auton A, et al. An integrated map of genetic variation from 1,092 human genomes. Nature. 2012;491(7422):56-65.

4 **Parry M, Rose-Zerilli MJJ, Ljungström V, Gibson J, Wang J, Walewska R, et al. Genetics and Prognostication in Splenic Marginal Zone Lymphoma: Revelations from Deep Sequencing. Clin Cancer Res 2015:[Epub ahead of print].**

**5** Bibikova M, Le J, Barnes B, Saedinia-Melnyk S, Zhou LX, Shen R, et al. Genome-wide DNA methylation profiling using Infinium (R) assay. Epigenomics 2009;1:177–200.

**6** Teschendorff AE, Marabita F, Lechner M, Bartlett T, Tegner J, Gomez-Cabrero D, et al. A beta-mixture quantile normalization method for correcting probe design bias in Illumina Infinium 450 k DNA meth- ylation data. Bioinformatics 2013;29:189–96.

**7** Assenov Y, Muller F, Lutsik P, Walter J, Lengauer T, Bock C. Compre- hensive analysis of DNA methylation data with RnBeads. 2013. Avail- able from: http://rnbeads.mpi-inf.mpg.de. Accessed February 1, 2013.

8 Oakes CC, Claus R, Gu L, Assenov Y, Hüllein J, Zucknick M et al. Evolution of DNA methylation is linked to genetic aberrations in chronic lymphocytic leukemia. Cancer Discov. 2014 Mar;4(3):348-61.

9 Oakes CC, Seifert M, Assenov Y, Gu L, Przekopowitz M, Ruppert AS, et al. DNA methylation dynamics during B cell maturation underlie a continuum of disease phenotypes in chronic lymphocytic leukemia. Nat Genet 2015, in press.

10 Kulis M, Heath S, Bibikova M, Queirós AC, Navarro A, Clot G, et al. Epigenomic analysis detects widespread gene-body DNA hypomethylation in chronic lymphocytic leukemia. Nat Genet. 2012 Nov;44(11):1236-42.

11 Queirós AC, Villamor N, Clot G, Martinez-Trillos A, Kulis M, Navarro A, et al. A B-cell epigenetic signature defines three biologic subgroups of chronic lymphocytic leukemia with clinical impact. Leukemia. 2015 Mar;29(3):598-605.
