# Supplementary material for: Longitudinal copy number, whole exome and targeted deep sequencing of 'good risk' IGHV-mutated CLL patients with progressive disease
Source: Leukemia. 2016 Feb 26;30(6):1301–10. doi: 10.1038/leu.2016.10 (PMC4861248; doi:10.1038/leu.2016.10)
Supplement: Supplementary Figure 1 [file leu201610x11.pdf]

A

B

C

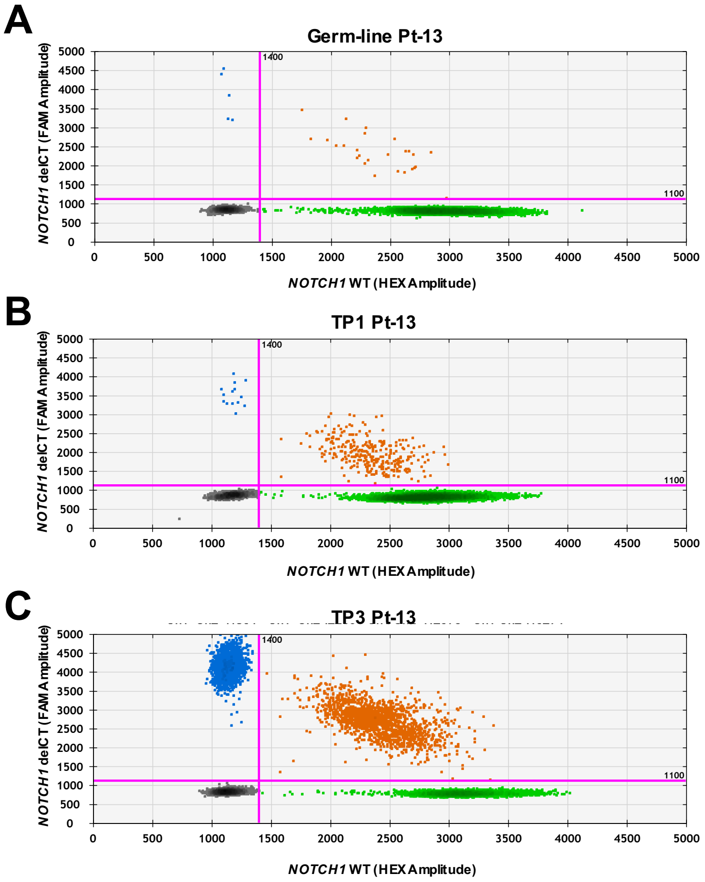

D

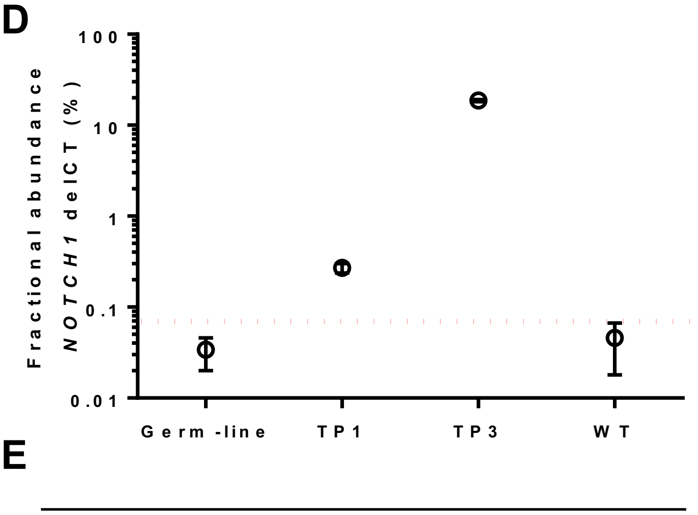

E

Sample	*NOTCH1* delCT droplets	*NOTCH1* WT droplets	Fractional abundance *NOTCH1* delCT (mean±95%CI)
Germ-line Pt-13	31	44312	0.034% (0.022-0.046)
TP1 Pt-13	323	48105	0.237% (0.24-0.30)
TP3 Pt-13	6771	23834	18.68% (18.26-19.09)
WT *NOTCH1*	18	26813	0.046% (0.024-0.067)

Supplemental Figure 1: Droplet-digital PCR analysis of the NOTCH1 delCT mutation in Patient-13 (5 x 50 ng of gDNA template/PCR). (A-C) X/Y-scattergraph of mutant positive (blue), mutant-wildtype double-positive (orange), wildtype positive (green) and negative droplets (black) Pinks lines are user-defined thresholds for each droplet cluster **(D)** Fractional abundance of mutant droplets in Germ-line, tumour TP1 and TP3 gDNA template from Patient-13 and a NGS NOTCH1 wildtype CLL control sample **(E)** Table displaying the droplet number for each sample in D and fractional abundance estimates (with 95% Confidence Intervals).
