# Supplementary material for: Longitudinal copy number, whole exome and targeted deep sequencing of 'good risk' IGHV-mutated CLL patients with progressive disease
Source: Leukemia. 2016 Feb 26;30(6):1301–10. doi: 10.1038/leu.2016.10 (PMC4861248; doi:10.1038/leu.2016.10)
Supplement: Supplementary Figure 2 [file leu201610x12.pdf]

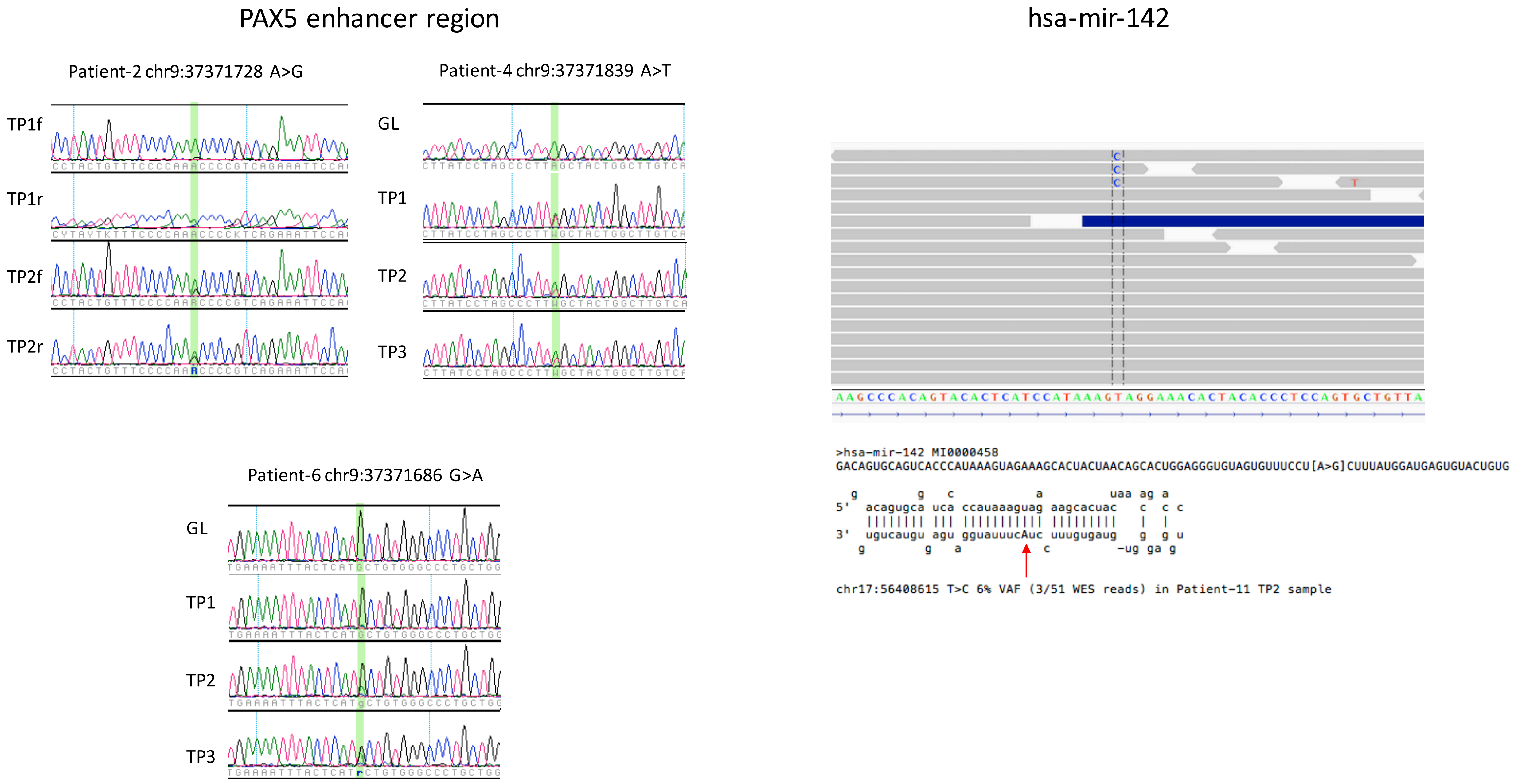
PAX5 enhancer region
Patient-2 chr9:37371728 A>G
TP1f
TP1r
TP2f
TP2r
Patient-4 chr9:37371839 A>T
GL
TP1
TP2
TP3
Patient-6 chr9:37371686 G>A
GL
TP1
TP2
TP3
hsa-mir-142
AAGCCCACAGTACACTCATCCATAAAGTAGGAAACACTACACCCTCCAGTGCTGTTA
>hsa-mir-142 MI0000458
GACAGUGCAGUCACCCAUAAAGUAGAAAGCACUACUAACAGCACUGGAGGGUGUAGUGUUUCCU[A>G]CUUUAUGGAUGAGUGUACUGUG
g g c a uaa ag a
5' acagugca uca ccauaaaguag aagcacuac c c c
3' ugucaugu agu gguauuucAuc uuugugaug g g u
g g a c -ug ga g
chr17:56408615 T>C 6% VAF (3/51 WES reads) in Patient-11 TP2 sample
