# Supplementary figures and images for: Longitudinal copy number, whole exome and targeted deep sequencing of 'good risk' IGHV-mutated CLL patients with progressive disease
Source: Leukemia. 2016 Feb 26;30(6):1301–10. doi: 10.1038/leu.2016.10 (PMC4861248; doi:10.1038/leu.2016.10)

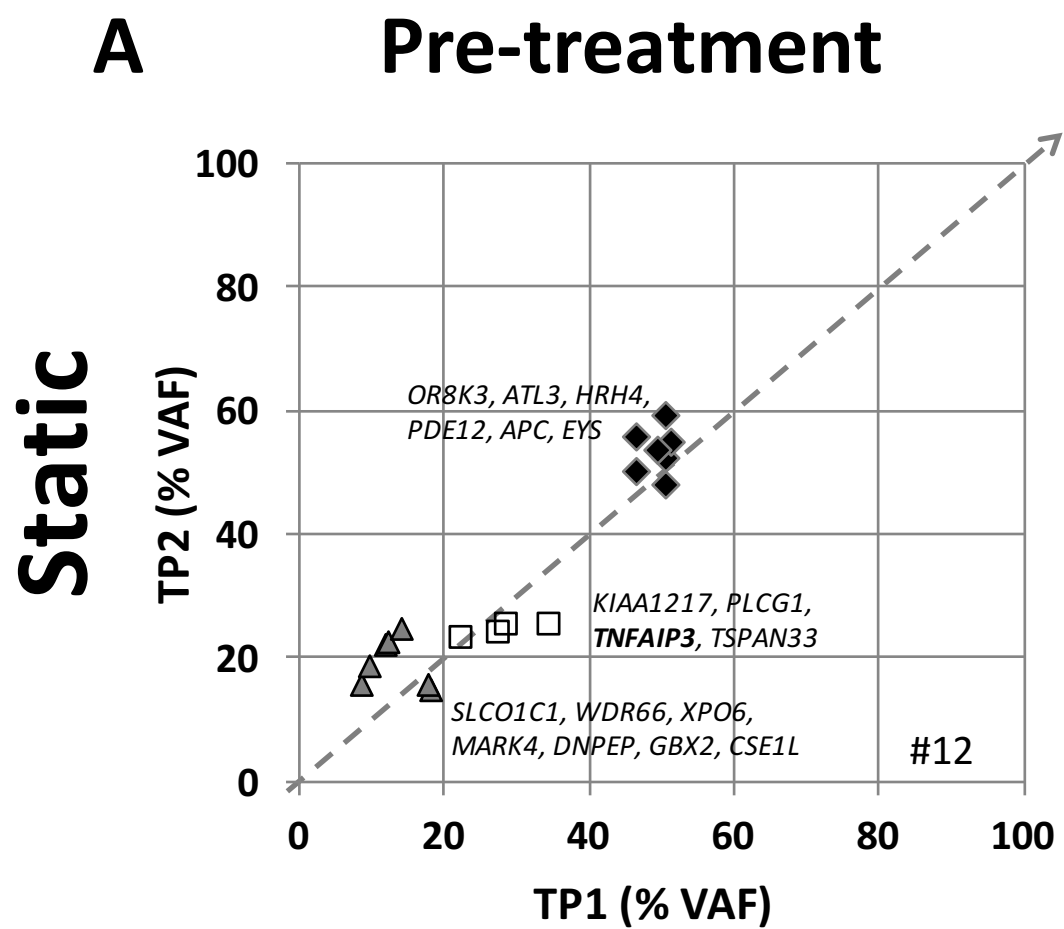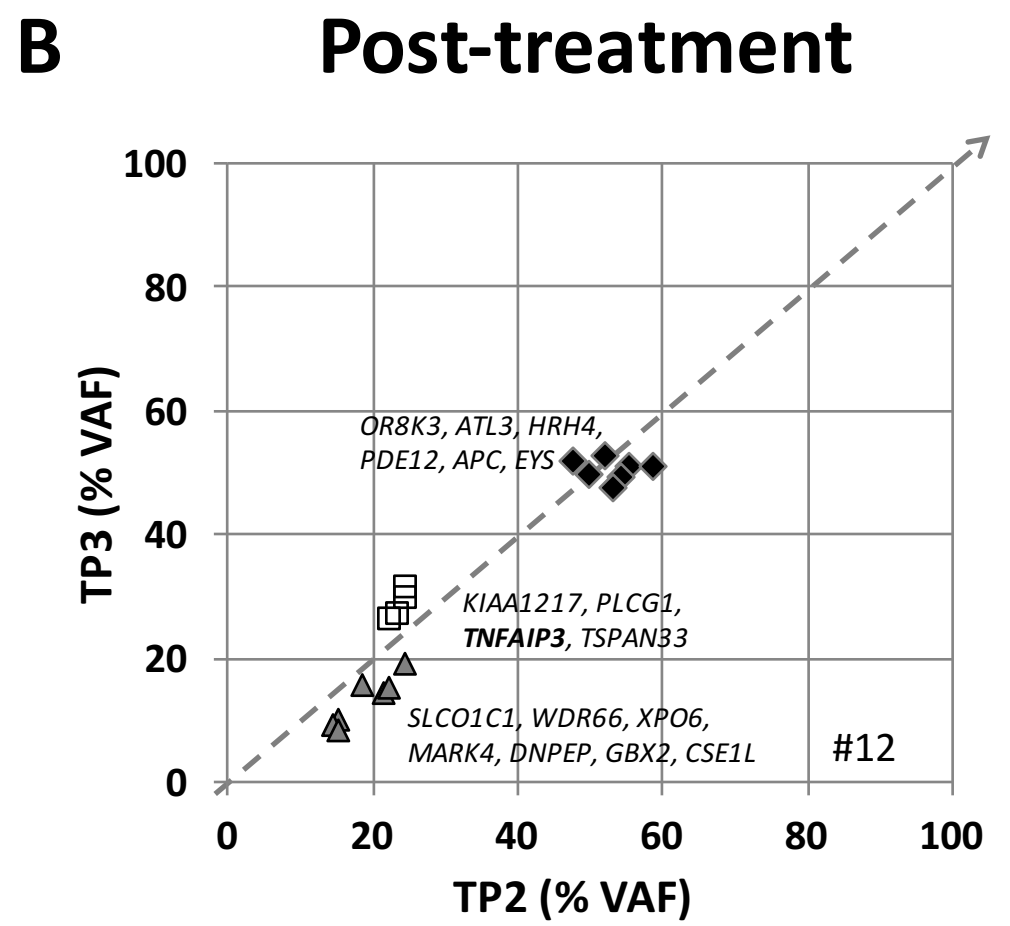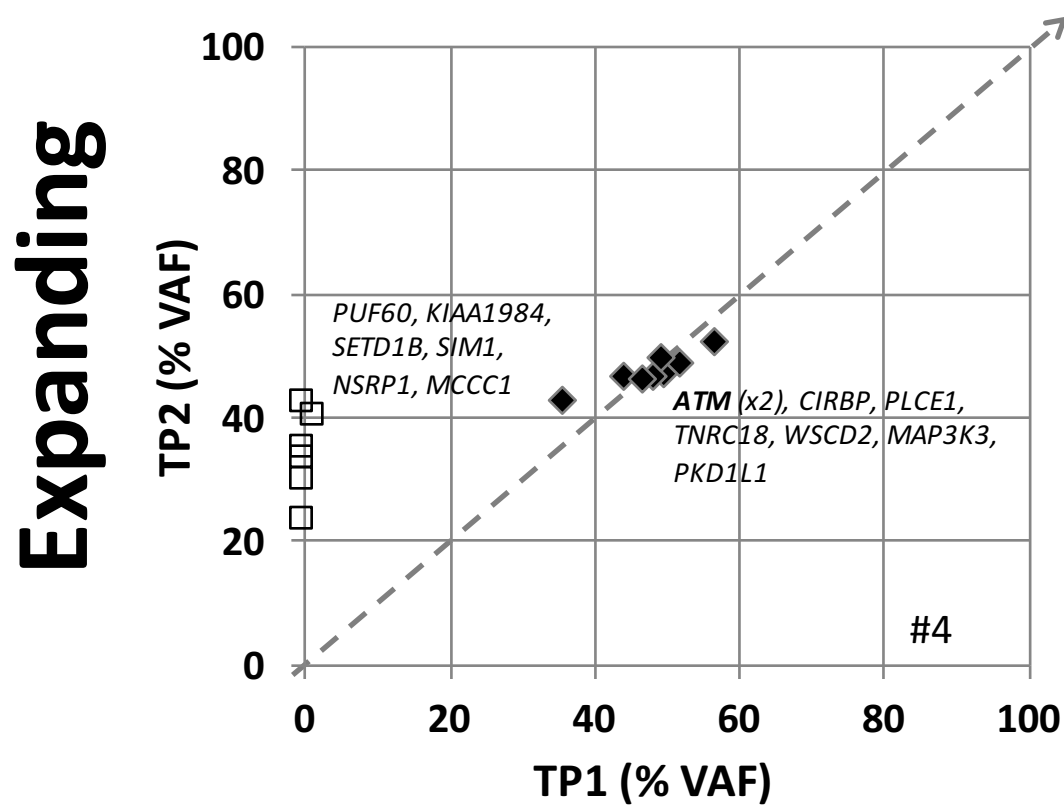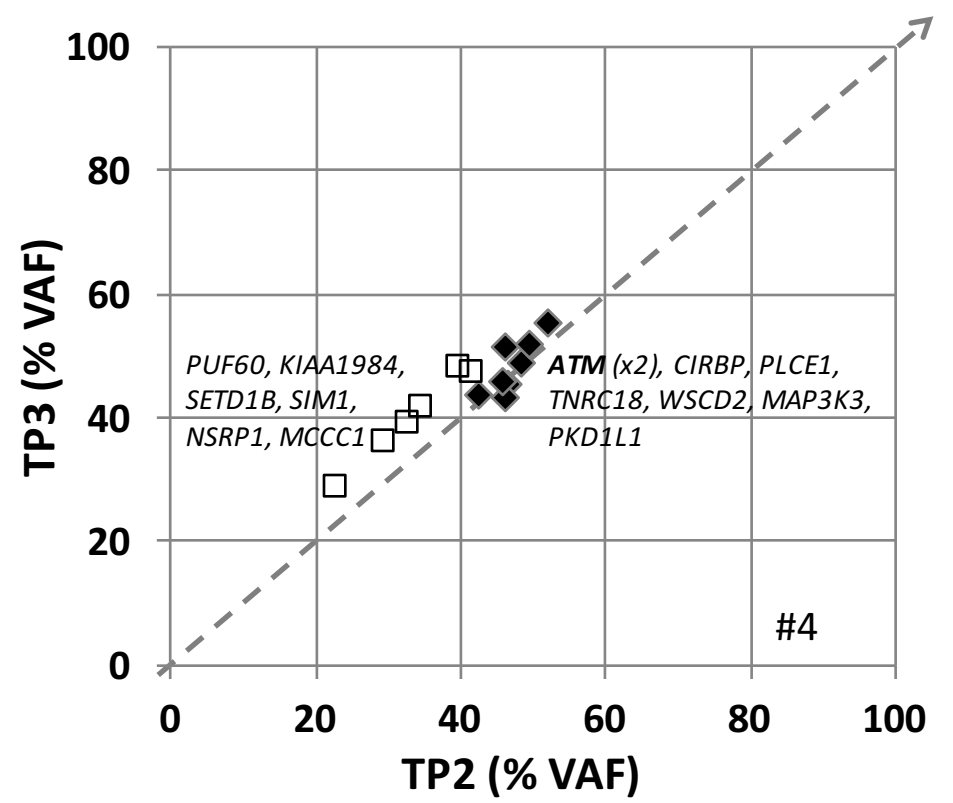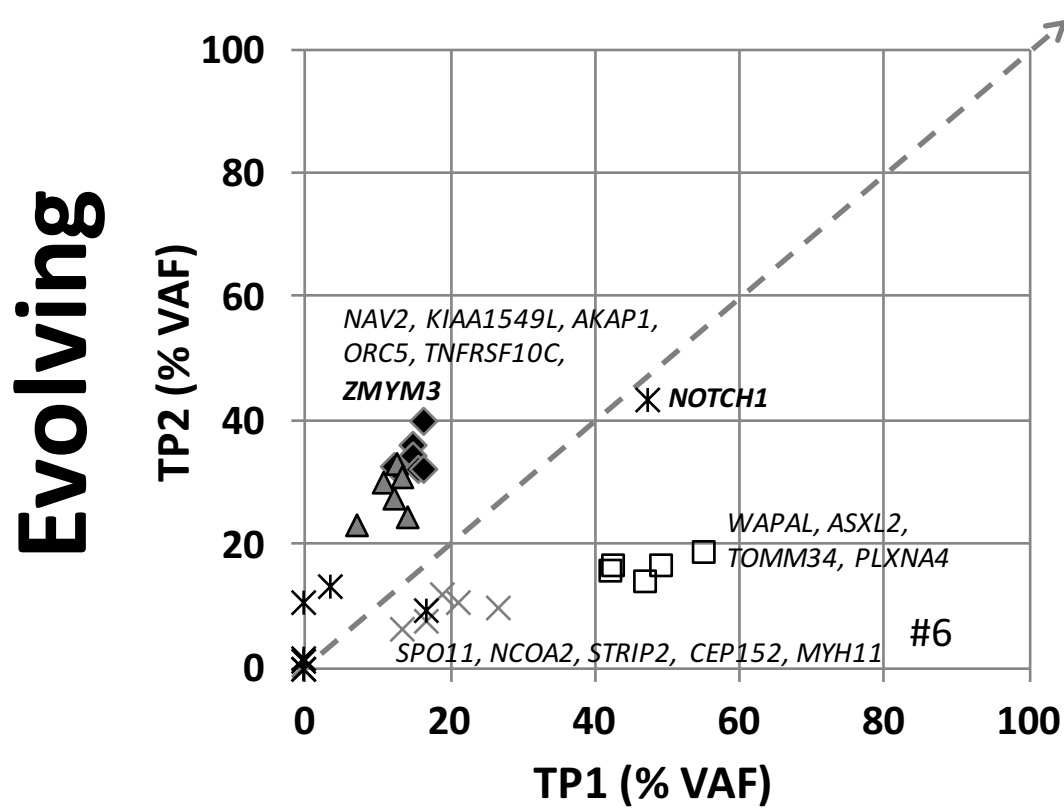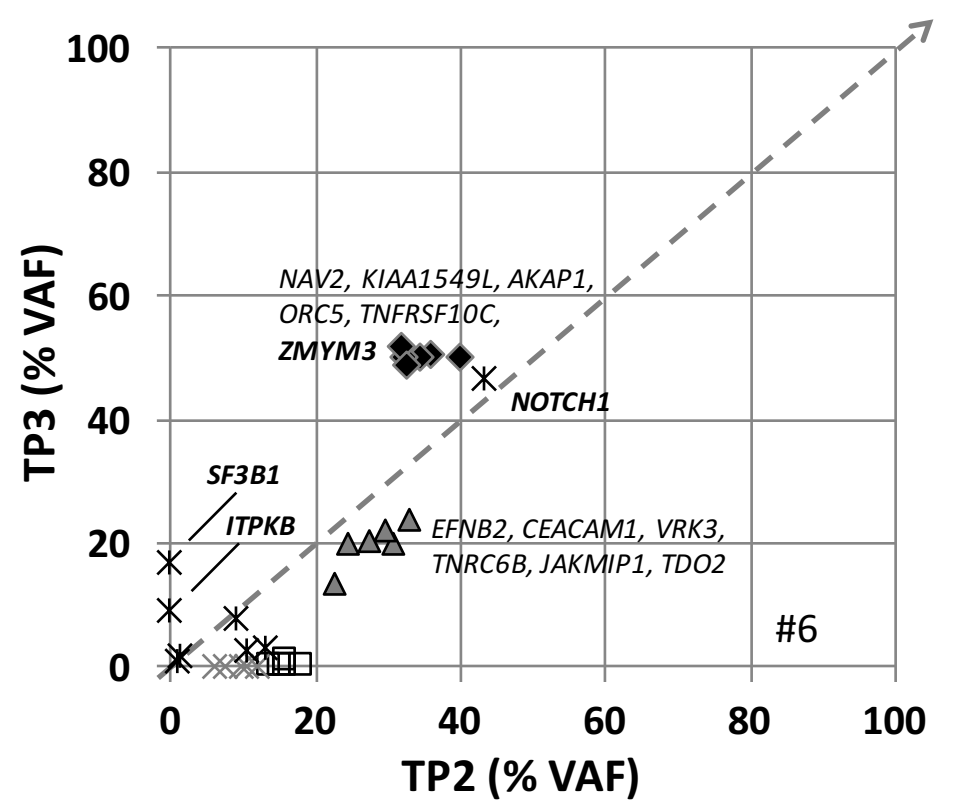

Supplement: Supplementary Figure 3 [file leu201610x13.pdf]

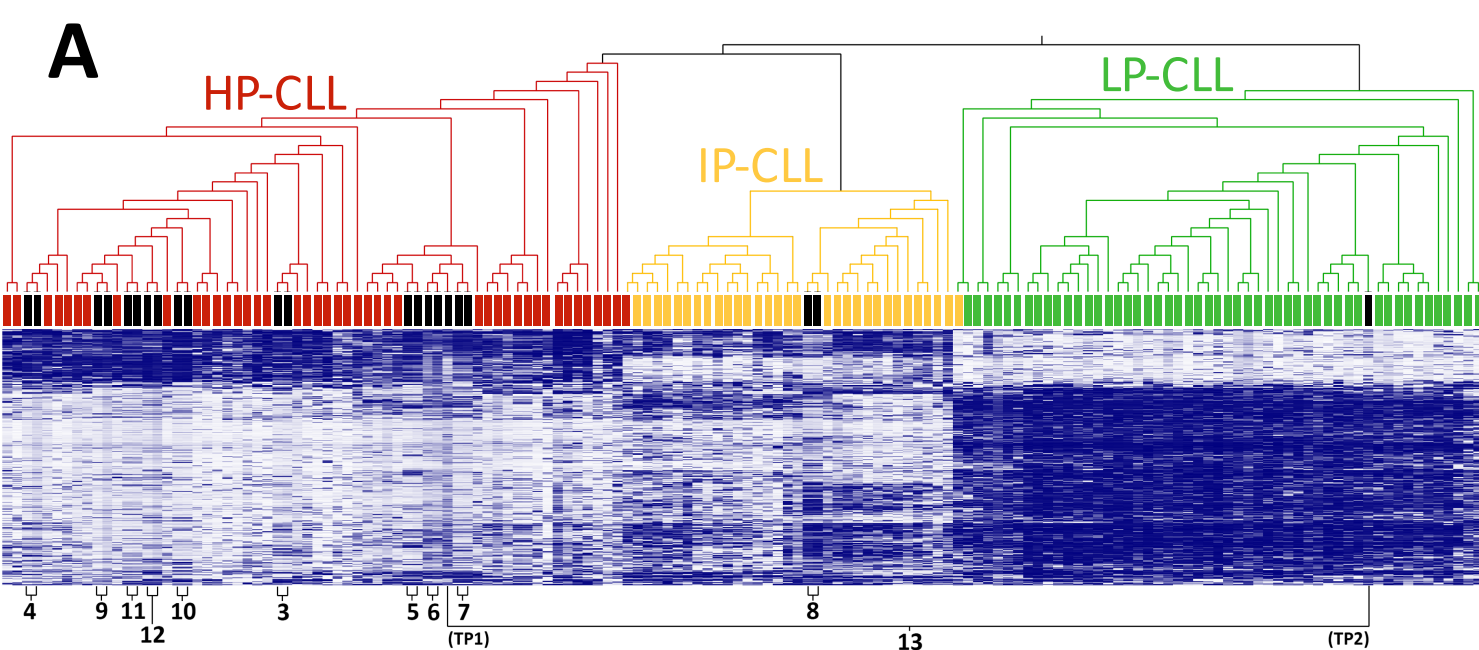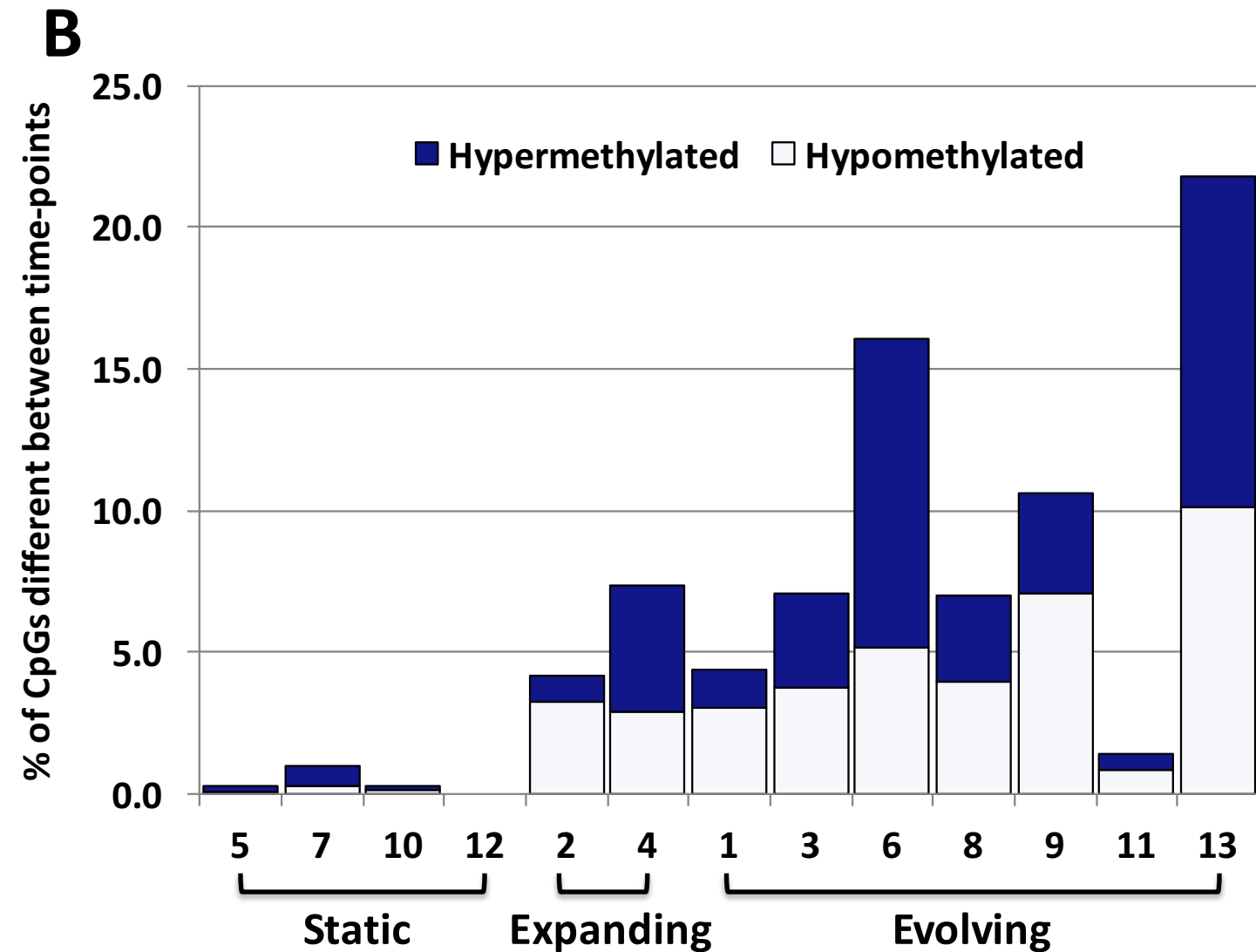

Supplement: Supplementary Figure 5 [file leu201610x15.pdf]
