# Supplementary material for: Longitudinal copy number, whole exome and targeted deep sequencing of 'good risk' IGHV-mutated CLL patients with progressive disease
Source: Leukemia. 2016 Feb 26;30(6):1301–10. doi: 10.1038/leu.2016.10 (PMC4861248; doi:10.1038/leu.2016.10)
Supplement: Supplementary Figure 4 [file leu201610x14.pdf]

Patient-1

SciClone analysis

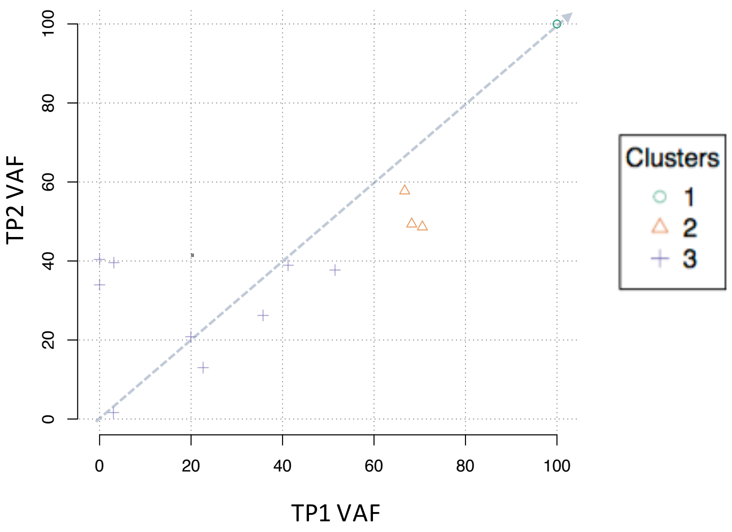

Gene	TP1.vaf	TP1.depth	TP2.vaf	TP2.depth	cluster	cluster.prob.1	cluster.prob.2	cluster.prob.3
TEAD4	20.33	1756	41.42	2636	0	NA	NA	NA
MAGEE1	100	568	100	2021	1	1.000000001	1.00E-09	1.00E-09
MAGEE1	100	2646	100	3590	1	1.000000001	1.00E-09	1.00E-09
MAGEC3	100	833	100	759	1	1.000000001	1.00E-09	1.00E-09
CHD2	66.71	1361	57.73	1639	2	1.00E-09	1.000000001	1.00E-09
DSEL	68.24	5478	49.36	7581	2	1.00E-09	1	2.11E-09
ARFGEF1	70.57	1695	48.62	2421	2	1.00E-09	0.999999996	6.08E-09
PLEKHH1	3.12	4904	39.58	3320	3	1.00E-09	1.00E-09	1.000000001
GLDN	35.74	3674	26.22	4924	3	1.00E-09	1.00E-09	1.000000001
PTPRM	51.48	2255	37.71	3023	3	1.00E-09	1.03E-09	1.000000001
FAM69C	0.3	6333	33.97	3232	3	1.00E-09	1.00E-09	1.000000001
PIGP;PIGP	19.95	972	20.84	1027	3	1.00E-09	1.00E-09	1.000000001
SLC34A1	3.02	563	1.63	429	3	1.00E-09	1.00E-09	1.000000001
GPRC6A	22.64	994	13.01	1330	3	1.00E-09	1.00E-09	1.000000001
THAP5	41.21	1541	38.92	2515	3	1.00E-09	1.00E-09	1.000000001
MLL3	0.73	7631	40.39	4511	3	1.00E-09	1.00E-09	1.000000001

Phylosub analysis

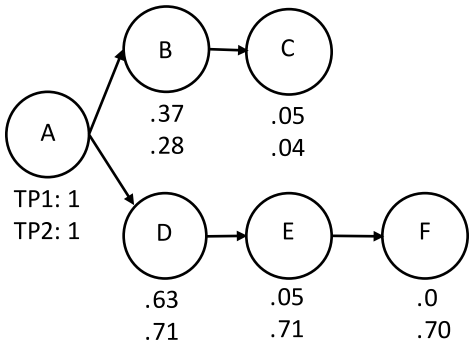

(A): *ARFGEF1; CHD2; DSEL; MAGEC3; MAGEE1; PTPRM*
(B): *PIGP; GPRC6A*
(C): *SLC34A1*
(D): *GLDN; TEAD4; THAP5*
(E): *PLEKHH1*
(F): *MLL3; FAM69C*

Patient-2

SciClone analysis

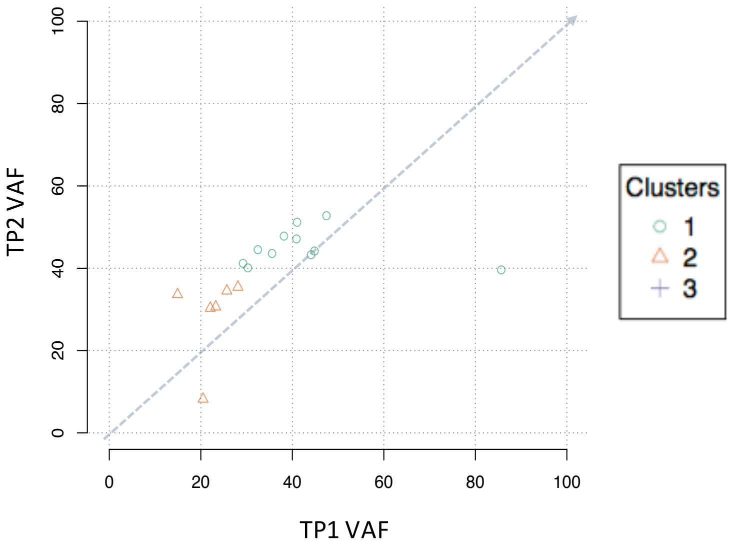

Gene	TP1.vaf	TP1.depth	TP2.vaf	TP2.depth	cluster	cluster.prob.1	cluster.prob.2
MYD88	40.91	5092	47.13	12896	1	0.999966759	3.32E-05
PFKFB4	41.03	1735	51.17	4884	1	0.999942136	5.79E-05
AMZ1	25.69	1106	44.51	2487	1	0.987121542	0.01287846
SMARCD3	32.46	1257	43.27	4063	1	0.99999661	3.39E-06
NOTCH1	22.06	1088	39.6	2992	1	1.000000001	1.00E-09
GPR158	44.09	2007	40.08	5469	1	0.8463529	0.153647102
EMC7	85.66	2817	43.58	6737	1	0.997480142	0.00251986
HS3ST6	30.29	1646	52.76	4663	1	0.999999745	2.57E-07
KCNJ2	20.49	613	47.81	1705	1	0.99971402	0.000285982
KRTAP13-2	23.26	2863	41.19	7152	1	0.854198591	0.145801411
SMCR7L	14.91	2287	44.19	5698	1	0.999998682	1.32E-06
NEK11	35.57	2180	34.49	4697	2	0.014007375	0.985992627
PPP1R9A	47.46	1818	30.29	3988	2	4.13E-05	0.999958723
PCDH8	38.17	683	8.22	1582	2	1.00E-09	1.000000001
CCDC168	29.23	2438	30.61	5002	2	7.72E-05	0.999922773
TCL1A	28.11	671	33.59	2054	2	0.005842621	0.994157381
NPTXR	44.86	157	35.43	113	2	0.072718217	0.927281785

Phylosub analysis

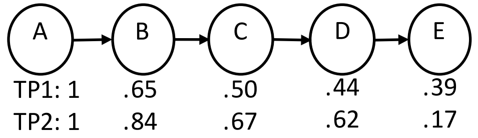

(A): *MYD88; SMCR7L; PFKFB4; KCNJ2; SMARCD3;HS3ST6*
(B): *AMZ1; NPTXR; EMC7; GPR158; KRTAP13-2*
(C): *NEK11*
(D): *TCL1A; CCDC168; PPP1R9A*
(E): *PCDH8*

Patient-3

SciClone analysis

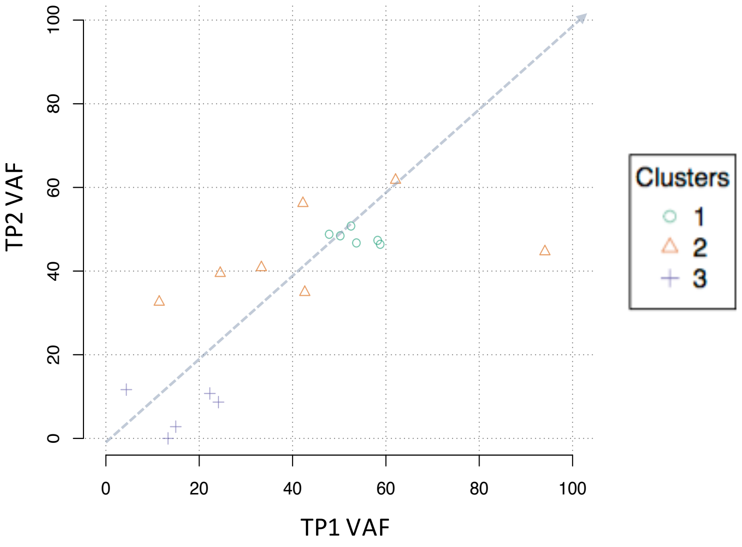

Gene	TP1.vaf	TP1.depth	TP2.vaf	TP2.depth	TP3.vaf	TP3.depth	cluster	cluster.prob.1	cluster.prob.2	cluster.prob.3
NID1	52.52	3081	50.77	3327	53.85	2503	1	0.983082402	0.0169176	1.00E-09
CACNB3	47.84	1674	48.8	2885	51.68	2419	1	0.945114696	0.054885306	1.00E-09
MYO1H	53.68	2155	46.73	1194	47.74	1552	1	0.960910152	0.03908985	1.00E-09
WFS1	58.78	3270	46.4	1347	57.33	1123	1	0.89180332	0.108196682	1.00E-09
PTPRK	50.22	2616	48.43	3302	50.94	3799	1	0.972683574	0.027316428	1.00E-09
PEX3	58.23	1642	47.34	2250	48.5	2602	1	0.935232938	0.064767064	1.00E-09
LRRIQ1	42.62	1720	34.92	1538	35.28	1284	2	4.15E-07	0.999999587	1.06E-09
ITGAE	24.5	5429	39.47	9734	45.06	9714	2	1.00E-09	0.999887291	0.000112711
CLDN14	62.07	1221	61.74	1406	65.88	1044	2	0.001048469	0.998951533	1.00E-09
LTF	11.43	11995	32.6	11653	41.98	10438	2	1.00E-09	0.972513157	0.027486845
TRAM1L1	33.32	1714	40.85	2610	41.05	2144	2	2.03E-08	0.999999841	1.42E-07
HIST1H3D	42.22	3245	56.19	2508	37.71	2814	2	0.000409301	0.999590701	1.02E-09
DCAF8L1	94.07	534	44.65	457	6.96	920	2	1.00E-09	1.000000001	1.00E-09
SELPLG	22.27	2420	10.74	4581	3.59	5487	3	1.00E-09	1.00E-09	1.000000001
CPNE2	14.97	2806	2.8	2536	0	2537	3	1.00E-09	1.00E-09	1.000000001
MAML3	13.32	7260	0	7259	12.8	7039	3	1.00E-09	1.00E-09	1.000000001
NMUR2	4.38	1781	11.66	1595	19.76	1245	3	1.00E-09	4.08E-07	0.999999594
PRUNE2	24.12	2629	8.67	2399	4.93	1623	3	1.00E-09	1.00E-09	1.000000001

Phylosub analysis

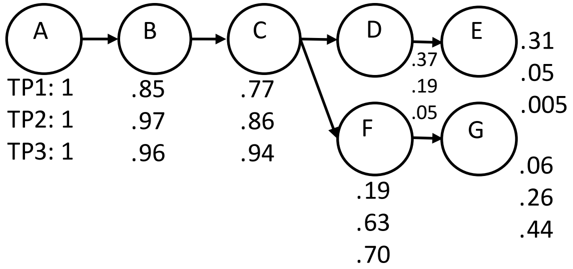

(A): *CACNB3; CLDN14; MYO1H; NID1; PEX3; PTPRK; WFS1*
(B): *HIST1H3D*
(C): *TRAM1L1; ITGAE; LRRIQ1*
(D): *DCAF8L1; PRUNE2;*
SELPLG
(E): *CPNE2*
(F): *LTF*
(G): *NMUR2*

Patient-5

SciClone analysis

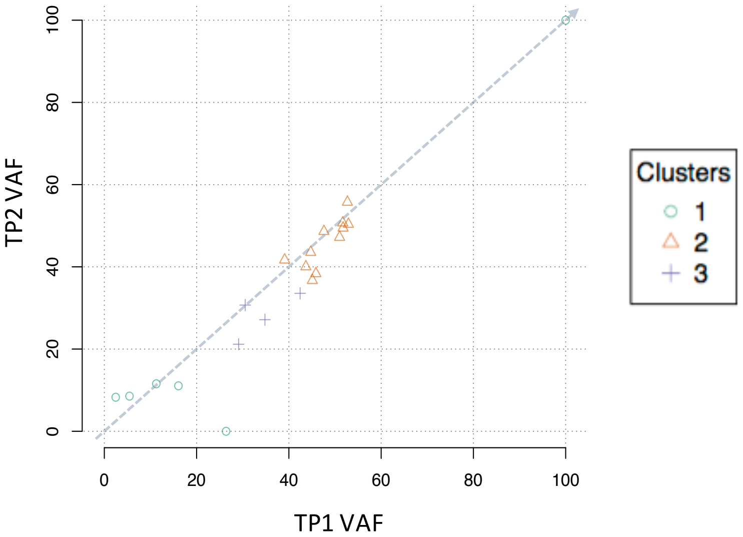

Gene	TP1.vaf	TP1.depth	TP2.vaf	TP2.depth	TP3.vaf	TP3.depth	cluster	cluster.prob.1	cluster.prob.2	cluster.prob.3
SLC8A1	2.46	3415	8.28	4396	14.9	3893	1	1.000000001	1.00E-09	1.00E-09
DNMT3B	26.39	557	0	577	25.89	819	1	1.000000001	1.00E-09	1.00E-09
SLC38A3	5.45	275	8.55	585	0	584	1	1.000000001	1.00E-09	1.00E-09
MAML3	11.26	5036	11.55	10390	15.06	7782	1	1.000000001	1.00E-09	1.00E-09
CDX4	16.06	685	11.06	1890	3.57	3866	1	1.000000001	1.00E-09	1.00E-09
NAP1L3	100	1550	100	3080	90.92	2678	1	1.000000001	1.00E-09	1.00E-09
JMJD1C	51.68	3071	50.71	6391	50.02	4404	2	2.33E-05	0.999976679	1.00E-09
METTL15	44.73	836	43.54	455	49.21	1262	2	7.65E-06	0.999992351	1.00E-09
PPP1R13B	45.09	3792	36.7	2191	45.88	4714	2	0.00011221	0.999887778	1.53E-08
BAIAP3	39.08	3306	41.69	8839	41.18	6282	2	0.000891572	0.999093286	1.51E-05
WSCD1	51.75	4891	49.45	11223	48.14	7316	2	1.24E-05	0.99998764	1.00E-09
ZNF132	47.6	5489	48.63	12881	50.13	8352	2	7.24E-06	0.999992764	1.00E-09
MORC1	43.68	5733	40.03	7844	42.13	8334	2	8.37E-05	0.999916097	2.44E-07
HERC6	45.86	443	38.36	576	46.97	688	2	3.20E-05	0.99996801	1.18E-09
MCCD1	50.99	1914	47.2	3756	45.95	2914	2	7.02E-06	0.999992987	1.00E-09
TLR4	52.91	5014	50.37	8974	50.64	6072	2	3.79E-05	0.999962074	1.00E-09
BRD3	52.69	3655	55.74	5427	50.1	4890	2	0.000500749	0.999499253	1.00E-09
USH2A	30.54	2338	30.7	4378	29.33	2578	3	0.000104937	1.00E-09	0.999895065
RANBP10	34.81	4211	27.14	5763	32.27	5277	3	0.000101744	1.00E-09	0.999898258
TMPRSS6	42.44	7205	33.56	15566	25.76	10423	3	0.001931863	1.05E-09	0.998068139
NFKBIE	29.11	5084	21.17	6665	25.34	6902	3	0.001600139	1.00E-09	0.998399863

Phylosub analysis

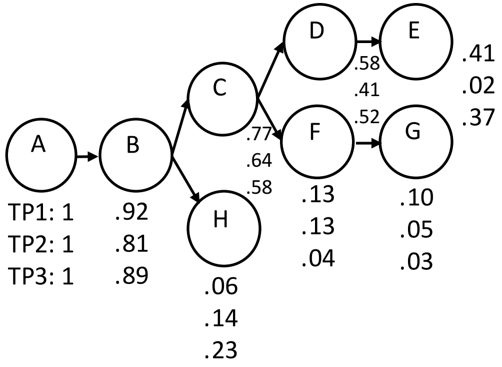

(A): *BRD3; JMJD1C; MCCD1; TLR4; WSCD1; ZNF132*
(B): *BAIAP3; HERC6; METTL15; MORC1; NAP1L3; PPP1R13B*
(C): *TMPRSS6; USH2A; RANBP10*
(D): *NFKBIE*
(E): *DNMT3B*
(F): *SLC38A3*
(G): *CDX4*
(H): *SLC8A1*

Patient-7

SciClone analysis

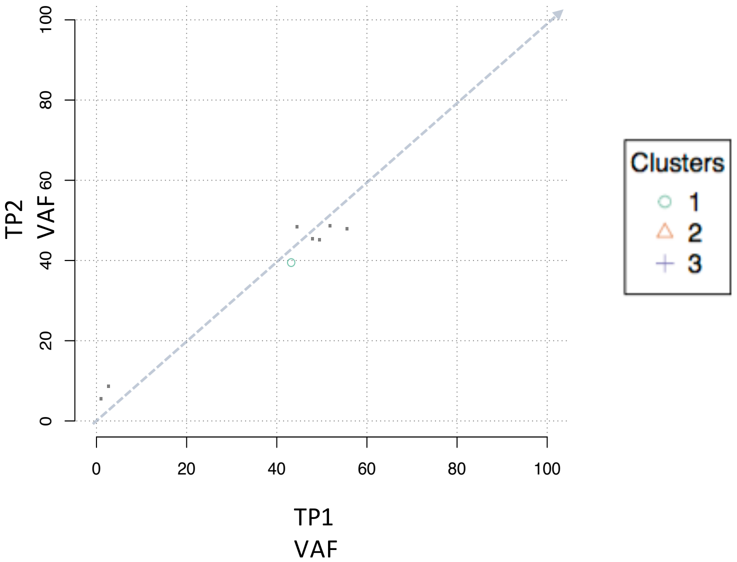

Gene	TP1.vaf	TP1.depth	TP2.vaf	TP2.depth	cluster	cluster.prob
RNPC3	2.7	185	8.64	2269	0	NA
AVPR1A	50.8	67	49.18	1580	0	NA
COTL1	1	500	5.49	3060	0	NA
MYH4	47.9	532	45.44	9018	0	NA
ERAL1	51.8	502	48.63	8116	0	NA
ZBTB7C	44.5	465	48.39	7264	0	NA
S1PR4	49.5	210	45.14	2984	0	NA
APBA3	42.6	49	49.4	2933	0	NA
MYO9B	55.6	568	47.94	16656	0	NA
N4BP3	43.2	132	39.48	2525	1	1.000000001

Phylosub analysis

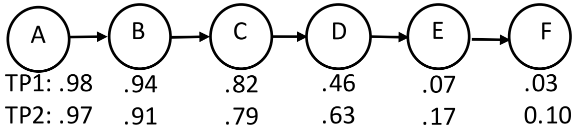

(A): *APBA3; AVPR1A; ERAL1; MYO9B; ZBTB7C*
(B): *S1PR4; MYH4*
(C): *N4BP3*
(D): empty
(E): *RNPC3*
(F): *COTL1*

Patient-8

SciClone analysis

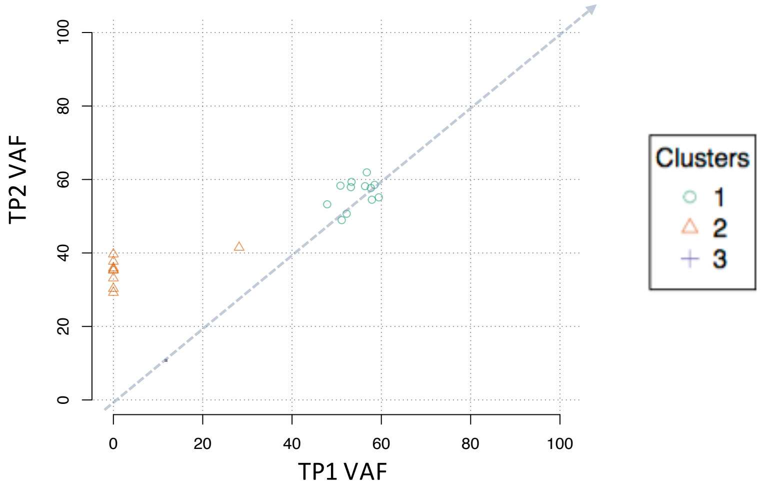

Gene	TP1.vaf	TP1.depth	TP2.vaf	TP2.depth	cluster	cluster.prob.1	cluster.prob.2
RNPC3	11.82	2107	10.74	3408	0	NA	NA
DCST1	56.35	4712	58.18	4966	1	1.000000001	1.06E-09
PIK3C2B	53.33	3321	59.35	3808	1	1.000000001	1.02E-09
PUS10	56.75	1621	61.94	1861	1	1.000000001	1.00E-09
LRP1B	59.43	1503	55.14	1630	1	0.999999995	7.08E-09
SF3B1	50.85	1233	58.32	1574	1	1.000000001	1.16E-09
GABRG1	52.23	1179	50.67	920	1	0.999996327	3.68E-06
JAK2	51.13	1172	48.94	1289	1	0.999913268	8.67E-05
DFNB31	58.48	5705	58.54	7229	1	1.000000001	1.05E-09
USP47	53.18	1213	57.92	2419	1	1.000000001	1.11E-09
IGLL5	57.66	4152	57.69	3855	1	1.000000001	1.13E-09
IGLL5	57.89	7304	54.5	7105	1	0.999999992	1.03E-08
LMF2	47.87	7708	53.24	8599	1	0.999999086	9.16E-07
HCN1	28.15	1002	41.49	1458	2	1.00E-09	1.000000001
VCAN	0	1066	35.25	1061	2	1.00E-09	1.000000001
SLC35G1	0.1	7116	37.68	7293	2	1.00E-09	1.000000001
ATHL1	0.05	5695	35.88	4142	2	1.00E-09	1.000000001
ARF6	0.04	5054	35.48	4377	2	1.00E-09	1.000000001
TP53	0.05	15586	29.21	10931	2	1.00E-09	1.000000001
OTOP3	0.08	13442	30.29	12172	2	1.00E-09	1.000000001
BFSP1	0.14	727	33.14	652	2	1.00E-09	1.000000001
UMODL1	0.29	6157	39.67	5415	2	1.00E-09	1.000000001

Phylosub analysis

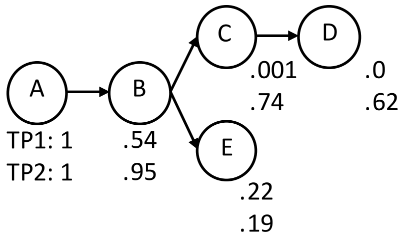

(A): *DCST1; DFNB31; GABRG1; IGLL5; JAK2; LMF2; LRP1B; PIK3C2B; PUS10; SF3B1; USP47*
(B): *HCN1*
(C): *ARF6; ATHL1; UMODL1; VCAN; SLC35G1*
(D): *TP53; BFSP1; OTOP3*
(E): *RNPC3*

Patient-10

SciClone analysis

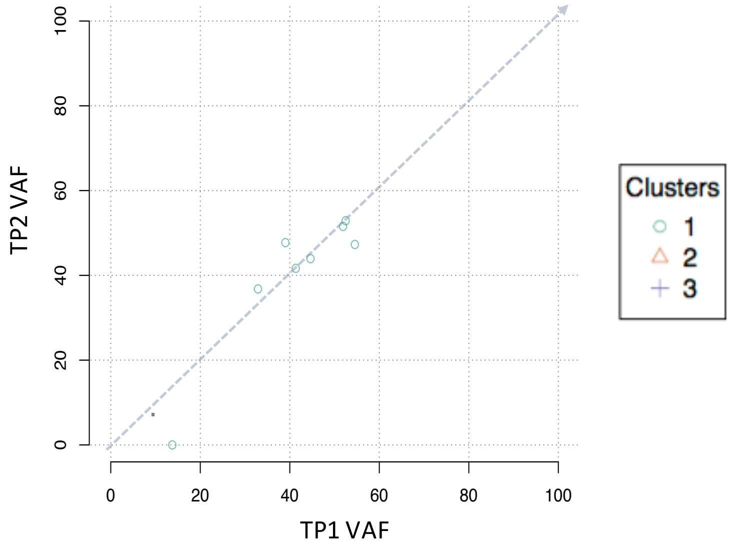

Gene	TP1.vaf	TP1.depth	TP2.vaf	TP2.depth	cluster	cluster.prob
PLEKHH1	9.43	3828	7.14	1625	0	NA
GLB1L	32.88	2707	36.8	4293	1	1.000000001
A4GNT	44.62	10399	43.94	8496	1	1.000000001
KLHL6	54.54	7451	47.29	5948	1	1.000000001
C9orf152	52.49	2738	52.9	2476	1	1.000000001
FAN1	39.01	2612	47.73	2458	1	1.000000001
ZNF423	51.89	5115	51.55	2826	1	1.000000001
ZNF793	41.34	8582	41.66	8041	1	1.000000001

Phylosub analysis

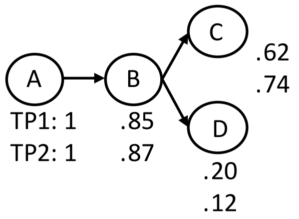

(A): *C9orf152; KLHL6; ZNF423*
(B): *A4GNT; ZNF793; FAN1*
(C): *GLB1L*
(D): *PLEKHH1*

Patient-11

SciClone analysis

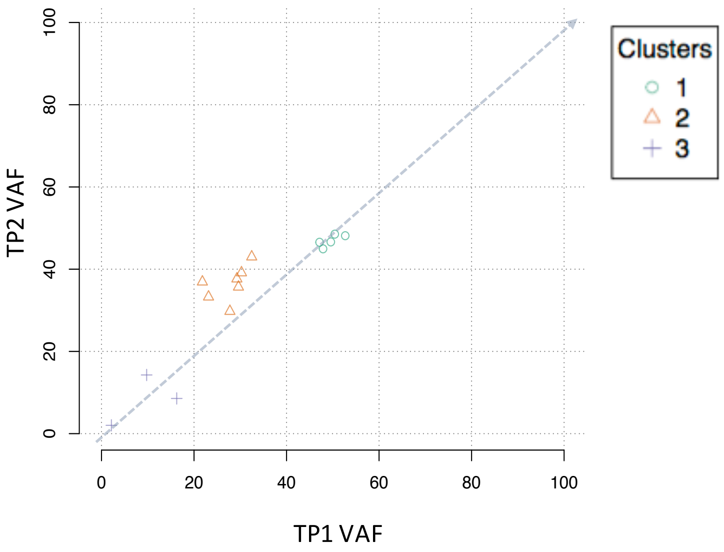

Gene	TP1.vaf	TP1.depth	TP2.vaf	TP2.depth	cluster	cluster.prob.1	cluster.prob.2	cluster.prob.3
OBSL1	47.89	4920	44.95	5003	1	0.999999966	3.64E-08	1.00E-09
CDHR4	49.57	5005	46.63	6142	1	1	1.66E-09	1.00E-09
ADRA1B	50.43	5243	48.52	5482	1	1.000000001	1.04E-09	1.00E-09
NCAPG2	52.69	6924	48.14	7921	1	1.000000001	1.00E-09	1.00E-09
OAS3	47.13	1935	46.55	3216	1	0.999999969	3.29E-08	1.00E-09
PRAMEF12	30.26	10314	39.14	11451	2	1.56E-09	1	1.00E-09
ITGA6	29.61	6008	35.67	5865	2	1.00E-09	1.000000001	1.00E-09
ARMC8	32.47	2513	43	1530	2	2.74E-06	0.999997262	1.00E-09
GP5	23.14	6335	33.26	7171	2	1.00E-09	1.000000001	1.00E-09
MICAL2	29.27	4045	37.65	5116	2	1.01E-09	1.000000001	1.00E-09
CUL5	21.81	2980	36.91	3278	2	1.00E-09	1.000000001	1.00E-09
DPH3P1	27.76	3635	29.76	3051	2	1.00E-09	1.000000001	1.00E-09
CADM2	9.76	5256	14.27	4919	3	1.00E-09	1.00E-09	1.000000001
OR4D5	16.26	2054	8.56	2336	3	1.00E-09	1.00E-09	1.000000001
CNGB1	2.14	10935	2.02	9752	3	1.00E-09	1.00E-09	1.000000001

Phylosub analysis

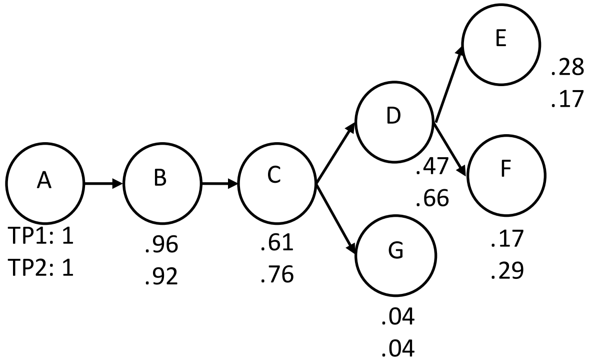

(A): *ADRA1B; CAAP1; NCAPG2*
(B): *CDHR4; OAS3; OBSL1*
(C): *PRAMEF12; ARMC8; ITGA6; MICAL2*
(D): *GP5; CUL5; DPH3P1*
(E): *OR4D5*
(F): *CADM2*
(G): *CNGB1*
