# Supplementary material for: Longitudinal copy number, whole exome and targeted deep sequencing of 'good risk' IGHV-mutated CLL patients with progressive disease
Source: Leukemia. 2016 Feb 26;30(6):1301–10. doi: 10.1038/leu.2016.10 (PMC4861248; doi:10.1038/leu.2016.10)
Supplement: Supplementary Legends [file leu201610x16.doc]

**Supplementary Table and Figure Legends**

Supplementary Tables:

Table S1: List of the 22 genes frequently mutated in CLL that were captured and resequenced by TDR

Table S2: Whole exome sequencing quality metrics and coverage statistics

Table S3: Targeted deep sequencing quality metrics and coverage statistics

Table S4: 786 mutations identified by whole exome sequencing

Table S5: Targeted deep sequencing validation of whole exome sequencing mutations

Table S6: Manually-curated established or putative mutations from the TDR data

Table S7: SNP6.0 copy number data

Table S8: Sequential targeted deep sequencing on patient 13

Table S9: Sequential IGHV analysis

Supplementary Figures:

Figure S1: Droplet-digital PCR analysis of the NOTCH1 delCT mutation in Patient-13. (A-C) X/Y-scattergraph of mutant positive (blue), mutant-wildtype double-positive (orange), wildtype positive (green) and negative droplets (black) Pinks lines are user-defined thresholds for each droplet cluster (D) Fractional abundance of mutant droplets in Germ-line, tumour TP1 and TP3 gDNA template from Patient-13 and a NGS NOTCH1 wildtype CLL control sample (E) Table displaying the droplet number for each sample in D and fractional abundance estimates (with 95% Confidence Intervals).

Figure S2: Non-coding mutation

(Left panel) Sanger-sequencing verified PAX5 enhancer region somatically-acquired mutations in three patients. Mutated base-pairs highlighted in green. Patients 2 and 6 have sub-clonal mutations at TP1. Patient-4 had a clonal mutation (overlapping base peaks) at TP1. (Right panel) A sub-clonal hsa-mir-142 mutation identified in whole exome sequencing (WES) data from patient 11 at disease progression (TP2). Mutated sequencing reads identified by blue C bases (6% VAF, 3/51 WES reads). Human mir-142 RNA sequence and mutation site in brackets. The red arrow indicates the position of the mutated base ( on the mature micro RNA hairpin molecule.

Figure S3: SciClone analysis of mutation clusters pre and post first-line therapy

XY scatter-graphs display the SciClone mutation clustering analysis performed on TDR datasets from sequential tumour time points in columns (A) before therapy and (B) after first-line treatment for three example patients displaying either a static (pt-12; treated with Chlor R. TEX13B data point not displayed as it resides on Chr X), an expanding (pt-4; BR) or an evolving (pt-6; Chlor) form of clonal dynamics prior to and after treatment. Data point symbols denote a distinct mutation cluster and the x=y line is displayed as a dashed arrow and denotes no change in the tumor purity-adjusted %VAF of mutation clusters between time points (clonal equilibrium). Selected gene symbols are displayed adjacent to its corresponding mutation cluster.

Figure S4: SciClone and Phylosub results for the remaining patients

(Left panel) XY scatter-graphs display the SciClone mutation clustering analysis performed on TDR datasets from sequential tumour time points TP1 (x-axis; first tumour sample) vs TP2 (y-axis; progression sample). Data point symbols denote a distinct mutation cluster and the x=y line is displayed as a dashed arrow and denotes no change in the tumor purity-adjusted %VAF of mutation clusters between time points (clonal equilibrium). Selected gene symbols are displayed adjacent to its corresponding mutation cluster. The table displays TDR %VAFs for each mutation, read depths, SciClone cluster assignment and probabilities (Right panel) Predicted phylogenetic tree structure (best model shown), with tumour time-point (TPn) population frequencies for each node from Phylo-sub analysis.

Figure S5: Programmed epigenetic subtypes and longitudinal stability of DNA methylation patterns

(A) Co-clustering heat-map of the top 1,000 most variable CpGs across all patient samples (black boxes and bracketed numbers) and 127 CLL cases (open boxes) previously assigned to one of the three primary epigenetic subtypes (12) termed: high (red dendrogram/boxes), intermediate (yellow dendrogram/boxes) and low (green dendrogram/boxes) programmed CLLs (HP-, IP-, and LP-CLLs, respectively). 12/13 cases clustered with HP-CLLs (patient-8 resides in IP-CLL group) and both time-points cluster adjacently, except for patient-13 where TP2 sample clusters with the LP-CLLs. Blue = hyper-methylated CpG, White = hypo-methylated CpG.

(B) Stacked bar chart of % of CpGs different between TP1 and TP2 450K DNA methylation datasets. Hyper- and hypo-methylation differences (Blue and white bars, respectively) of greater than 10% (out of a total of 459,625 CpGs analyzed) were tallied per case and were adjusted for differences in tumor cell content and copy number changes between samples. Patients are grouped by their genomic evolution status from SciClone analysis (Static, Expanding and Evolving). Patient-11 has a small % methylation difference probably corresponding to the evolution of only a minor sub-clonal mutation cluster at TP2 (cluster #3; See supplementary figure S2).
